# Comparison of out-of-plane short axis with in-plane long axis for ultrasound-guided radial arterial cannulation: A systematic review with trial sequential analysis of randomised controlled trials

**DOI:** 10.3389/fcvm.2022.983532

**Published:** 2022-10-12

**Authors:** Xia-xuan Sun, Meng Lv, Wen-ya Du, Yi Liu, Haixia Zhang, Yue-lan Wang

**Affiliations:** ^1^Department of Anesthesiology, The First Affiliated Hospital of Shandong First Medical University & Shandong Provincial Qianfoshan Hospital, Shandong Institute of Anesthesia and Respiratory Critical Care Medicine, Jinan, China; ^2^Shandong First Medical University & Shandong Academy of Medical Sciences, Jinan, China

**Keywords:** cannulation, catheterisation, long-axis in-plane, radial artery, short-axis out-of-plane, ultrasound guidance

## Abstract

**Background:**

It is controversial whether the short-axis out-of-plane or long-axis in-plane approach is a better needling technique for ultrasound-guidance radial artery cannulation. We aimed to compare the efficacy and safety of the two approaches for ultrasound-guided radial artery cannulation.

**Methods:**

A systematic search of Medline, Embase, the Cochrane Library, and Web of Science for relevant articles published until 1 May 2021 was conducted. Randomised controlled trials comparing the long-axis in-plane with short-axis out-of-plane approaches were included. Review Manager software version 5.4, STATA version 14.2, and trial sequential analysis (TSA) version 0.9.5.10 Beta were used for statistical analysis. Risk of bias and methodological quality of all studies included in this review were assessed according to the Cochrane Collaboration tool for the risk of bias. Subgroup analyses and meta-regression were performed to explore sources of heterogeneity.

**Results:**

The rate of cannula insertion success on the first attempt was similar between the short-axis out-of-plane and long-axis in-plane approaches (RR = 1.03; 95% CI: 0.83 to 1.28; *P* = 0.79; *I*^2^ = 83.0%). No significant differences were observed in total time to successful cannulation between the two approaches (MD = –3.9; 95% CI:-18.30 to 10.49; *P* = 0.6; *I*^2^ = 97%). However, the required information size for the success rate of the first attempt and total time to successful cannulation was not reached.

**Conclusion:**

It remains inconclusive whether short-axis out-of-plane is a better choice for radial arterial cannulation than the long-axis in-plane approach. Inexperienced operators may need more attempts and longer ultrasound location time with the short-axis out-of-plane technique.

**Systematic review registration:**

[https://www.crd.york.ac.uk/prospero/], identifier [CRD42021236098].

## Introduction

Radial arterial cannulation is a frequent and important procedure performed in many clinical settings, including the intensive care unit (ICU), emergency department, and operating room. It allows continuous blood pressure monitoring and numerous arterial blood sampling. Traditionally, radial artery catheterisation has been performed by the guidance of pulse palpation and anatomical knowledge; however, the insertion of catheters may be challenging in some circumstances. Risk factors associated with cannulation failure include obesity, hypotension, oedema, atherosclerosis, atrial fibrillation, and arterial scarring. In small children and infants, smaller arterial diameter is also a risk factor. In an attempt to reduce the rate of cannulation failure, the use of ultrasound guidance has gained significant popularity. Previous studies have confirmed that the use of ultrasound guidance is a well-validated adjunct for arterial cannulation in both children and adults (1–5). The American Society of Echocardiography and Society of Cardiovascular Anesthesiologists have recommended ultrasound as an effective rescue technique for arterial access (6). The European Society of Anesthesiology recommends the use of ultrasound guidance for radial artery catheterisation in all children and adults with hypotension, hypovolaemia, haemodynamic variables, vascular diseases, and small arteries with weak pulses (7).

However, there are two commonly used techniques for ultrasound-guided arterial cannulation: short-axis out-of-plane (SA-OOP) and long-axis in-plane (LA-IP). Both SA-OOP and LA-IP can improve the success rate of the first attempt and reduce the catheterisation time and rate of complications. A previous systematic review also suggested that there was no significant difference in total success rate between the two approaches in vascular catheterisation (8). Recently, several modified SA-OOP techniques have emerged, including the dynamic needling tip position technique and special marker in the transducer (9, 10). However, it remains controversial which approach is preferable in ultrasound-guided radial artery cannulation. Therefore, this systematic review aimed to compare the efficacy and safety of the two ultrasound-guided approaches for radial artery cannulation.

## Methods

### Study registration and reporting

The methods and analysis plan were registered in the International Prospective Register of Systematic Reviews (PROSPERO) database (CRD42021236098). This systematic review and meta-analysis was performed according to the Preferred Reporting Items for Systematic Reviews and Meta-Analysis (PRISMA) guidelines (11).

### Search strategy

Two investigators (X-xS and W-yD) independently searched Medline, Embase, Cochrane Library, and Web of Science databases from inception to May 1st, 2021. The electronic search strategy (combined MeSH terms and key words) included “Radial Artery,” “Catheterization,” “catheterization, peripheral,” “Catheters,” “Vascular Access Devices,” “Punctures,” “Ultrasonography,” “Ultrasonics,” “Ultrasonic Waves,” and “ultrasonography, doppler.” In addition, a manual search was performed for references to relevant articles, reviews, and meta-analyses. A detailed search strategy is provided in the Supplementary Material 1.

### Inclusion and exclusion criteria

Two reviewers (W-yD and X-xS) independently assessed potentially relevant studies for inclusion in the systematic review. For a study to be included, it had to be a randomised controlled trial (RCT) reporting on the SA-OOP versus LA-IP for ultrasound-guided radial arterial cannulation. Studies investigating other approaches (oblique versus longitudinal axis/in-plane approaches or dynamic needle tip positioning technique versus palpation technique) for ultrasound-guided radial artery cannulation were excluded.

### Data extraction

Two reviewers (X-xS and W-yD) independently extracted the data from each article that met the inclusion criteria. Any disagreement was resolved by discussion until consensus was reached or by consulting a third author. The following variables were extracted: author, publication time, mean age of the study population, body weight, sex, manufacturer of the ultrasound device, clinical settings of the included studies, sample size of the included study, size of arterial cannula, model of ultrasound probe, parameter settings of the probe, administration of local anaesthesia before arterial puncture, whether arterial catheterisation was performed after general anaesthesia, extension of patient hand position, rate of cannula insertion success on the first attempt, total cannulation time, potential complications, ultrasonic location time, cannulation time, anteroposterior arterial diameter, and skin-to-artery distance.

### Risk of bias and evidence quality assessment

Two reviewers (X-xS and W-yD) independently assessed the risk of bias and methodological quality of the included studies using the Cochrane Collaboration’s tool for randomised controlled trials. Items were evaluated in three categories: low risk of bias, unclear bias, and high risk of bias. The following domains were evaluated: random sequence generation (selection bias), allocation concealment (selection bias), blinding of participants and personnel (performance bias), incomplete outcome data (attrition bias), selective reporting (reporting bias). Risk of bias assessment for included studies is available in Supplementary Material 2. We evaluated each combined outcome in this systematic review and meta-analysis using the Grade of Recommendations Assessment, Development, and Evaluation (GRADE). We categorized evidence quality of the outcomes into four levels, from very low to high based on five domains, the risk of bias, inconsistency, indirectness, imprecision, and other considerations (Supplementary Table 3). Any differences were resolved by discussion until consensus was reached.

### Statistical analysis

Statistical analyses were performed using STATA (MP 14.2), trial sequential analysis (TSA) (Version 0.9.5.10 Beta, Copenhagen Trial Unit, Center for Clinical Intervention Research, Rigshospitalet, Copenhagen, Denmark), and Review Manager 5.4 software (Nordic Cochrane Centre, Copenhagen, Denmark). Dichotomous variables are presented as risk ratios (RRs) or odds ratios (ORs). Continuous variables are presented as weighted or standardised mean differences. In our studies, the confidence interval (CI) will be established at 95%, and *P*-value < 0.05 will be considered statistically significant.

Statistical heterogeneity among the included studies was assessed using the χ^2^ (Cochran’s Q) and *I*^2^ methods. Heterogeneity was considered according to the Cochrane Handbook as follows: not important (0–40%), moderate heterogeneity (30–60%), substantial heterogeneity (50–90%), and considerable heterogeneity (75–100%). According to *Cochrane Handbook*, a fixed-effect model was used when there was no statistical heterogeneity. When there was heterogeneity that cannot be explained, one analytical approach was to incorporate it into a random-effects model (12). Sources of heterogeneity were explored using subgroup and meta-regression analyses. Subgroup analysis was performed based on the different experience levels of the staff and type of SA-OOP technique. Meta-regression analyses were performed to investigate the association between each outcome and other factors, the anaesthetic status of the patients and local anaesthesia before cannulation. Sensitivity analysis was performed to ascertain the robustness of the results. Sensitivity analysis was performed based on the characteristics of participants and analysis methods (Supplementary Table 4).

Outcomes with double-arm-zero-events were excluded, even though treatment was not treated as “non-informative” (13). In multiple-arm studies, means and standard deviations (SDs) of different groups were combined into a single group using an algorithm described by Cochrane Collaboration (12). Means and SDs of the sample were estimated from the third quartile, first quartile, median, and size of the sample (14, 15). Publication bias was assessed using funnel plots, Begg’s and Egger’s tests.

### Trial sequential analysis

This systematic review and meta-analysis only included seven studies, which may have resulted in type I errors due to an increased risk of random errors resulting from sparse studies and data. To assess the risk of type I errors, we applied TSA, a procedure that combines an estimation of information size (cumulated sample size of included trials) with an adjusted threshold for statistical significance in cumulative meta-analyses. When the cumulative Z-curve crosses the trial sequential monitoring boundary or enters the futility area, a sufficient level of evidence for the anticipated intervention effect may have been reached, and no further trials are needed. In the converse situation, the evidence to reach a conclusion is insufficient, and further trials are needed to confirm the results. For this TSA, we estimated the required information size (RIS) using α = 0.05 (two sided), β = 0.20 (power = 80%), control event proportion (success rate of first attempt) of 68% calculated from the long-axis group, and a relative risk reduction of 20% in outcomes. With regard to total cannulation time, the RIS was estimated using α = 0.05 (two sided), β = 0.20 (power = 80%), and user-defined mean difference between two groups of –10. TSA was conducted using TSA software.

## Results

### Literature search results

**FIGURE 1:**
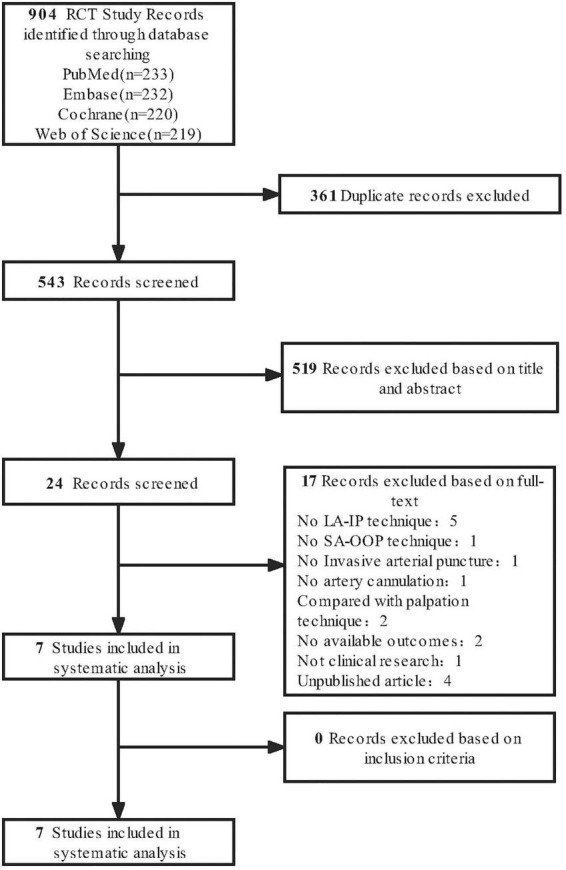
Flowchart of study selection.

The initial systematic literature search yielded 904 studies from different databases, of which 24 were included in the full-text review. Seven were retrieved for the final analysis. These articles were selected for retrieval based on a review of potentially eligible titles and abstracts. All seven trials met the inclusion criteria (Figure 1).

### Characteristics of included studies

The study characteristics are summarised in Table 1. The included studies (six adult studies and one paediatric study) were published between 2013 and 2020 and comprised 826 patients. The sample sizes of the studies ranged from 84 to 163. The baseline characteristics of the patients are summarised in Table 1. There were no significant differences in baseline variables between the groups. All staff had experience in ultrasound-guided radial artery catheterisation; however, the experience of the staff who performed arterial cannulation differed among trials. Only one study included a population of children younger than 5 years of age, (16) and one study included both ICU-admitted and surgical patients (17). All seven studies compared SA-OOP with LA-IP. Two studies used a modified SA-OOP technique with a sterile marker on the midpoint of the ultrasound probe (9, 17). Two studies used a dynamic needle tip positioning technique in the SA-OOP group (10, 16). There were no significant differences in the skin-to-artery distance and anteroposterior arterial diameter between the SA-OOP and LA-IP groups.

**TABLE 1:** Characteristics of included studies.

Study	Sample size	Age (SA-OOP/LA-IP)	Sex (M/F)	Weight	Ultrasound device (machine)	Ultrasound device (probe)
Berk et al. (21)	108	56 ± 1/54 ± 2	23/31; 30/24	78 ± 18; 76 ± 16	Esaote My Lab 30, Ultrasound Machine, Florence, Italy	NA
Quan et al. (9)	163	49.2 ± 08.1/46.1 ± 07.9	59/22; 64/18	76.4 ± 12.2/72.1 ± 10.5	ultrasound system (Terason2000 +, Terason, Burlington, MA, USA)	NA
Sethi et al. (17)	150	59.5 ± 8.2/57.7 ± 7.6	46/29; 41/34	62.8 ± 11.6/64.6 ± 12.2	ultrasound system (Sonosite^®^ MicroMaxx^®^ Ultrasound System, Sonosite INC., Bothell, WA, USA)	Hockey-stick shaped ultra-sonic probe (MicroMaxx^®^ SLA/13–6 MHz, Sonosite INC., Bothell, WA, USA)
Song et al. (16)	101	5.6 ± 3.7/4.3 ± 3.0 (month); 3.4 ± 2.8/3.3 ± 1.2 (year)	72/29	7.4 ± 2.1;8 ± 2.3 (infant); 14.0 ± 3.3;14.8 ± 3.9 (paediatrics)	ultrasound (LOGIQ e; GE Healthcare, Wauwatosa, Wisconsin, USA)	Hockey stick transducer (i12L-RS; GE Healthcare, Wauwatosa, Wisconsin, USA)
Abdalla et al. (19)	126	55 ± 11/59 ± 9	–	84 ± 32/84 ± 31	Toshiba Xario, Japan, PLT 805AT transducer	NA
Arora et al. (20)	84	54.101 ± 7.17/56.69 ± 14.82	–	–	iE33 Philips ultrasound machine	Linear LI5-7io probe
Nam et al. (10)	136	64.3 ± 13.0/63.6 ± 13.3	43/27; 33/30	64.3 ± 14.9/63.2 ± 12.2	ultrasound system, iE33; Philips	Linear “hockey stick” probe (L15-7io; Philips)

**Study**	**Clinical setting**	**Anaesthesia status**	**Hand posture**	**Local anaesthesia**	**Size of cannula**	**Ultrasound setup**

Berk et al. (21)	Operating room	Following general anaesthesia induction	Dorsiflexion ∼45°	Without local anaesthesia	Standard 20-G 1.v. cannula (Lakhani Medicare Pvt., Ltd., Haryana, India)	18 MHz frequency, 2 cm depth
Quan et al. (9)	Operating room	Following general anaesthesia induction	Positioned in dorsiflexion and fixed to the roll	Local anaesthesia (0.2 mL, 2% lidocaine)	BD	18 MHz frequency, 2 cm depth
Sethi et al. (17)	Operating room	Following general anaesthesia induction	Positioned in dorsiflexion and fixed to the roll	NA	Standard 20-G arterial cannula (BD Venflon™ Pro Safety Shielded IV cannula system)	NA
Song et al. (16)	Operating room	Following general anaesthesia induction	Extended over a roll and dorsiflexed for best visualisation of the artery	Local anaesthesia (0.2 mL, 2% lidocaine)	NA	NA
Abdalla et al. (19)	Operating room or ICU	NA	Moderate dorsiflexion of the wrist with a towel under its dorsal aspect	NA	Catheter needle system (Leadercath Arterial; Vygon, UK)	8 MHz frequency; depth, 3 cm
Arora et al. (20)	Operating room	Before general anaesthesia	Positioned in dorsiflexion at approximately 45° and fixed over a roll	0.2–0.5 mL 2% lidocaine	20-G catheter (Jelco IV; Smiths Medical, Dublin, OH, USA)	NA
Nam et al. (10)	Operating room	Prior to general anaesthesia induction	Wrist dorsiflexed at 45° and taped on the armrest	< 1 mL of 2% lidocaine	20-gauge, 1.16-inch intravenous catheter (BD angiocath Plus™; Becton Dickinson, Franklin lakes, NJ, USA)	NA

S-Bp, systolic pressure; D-Bp, diastolic pressure.

Publication bias was evaluated using funnel plots, Begg’s and Egger’s tests, which revealed no significant publication bias for the rate of cannulation success on the first attempt (Supplementary Material 5).

### Risk of bias and methodological quality

**FIGURE 2:**
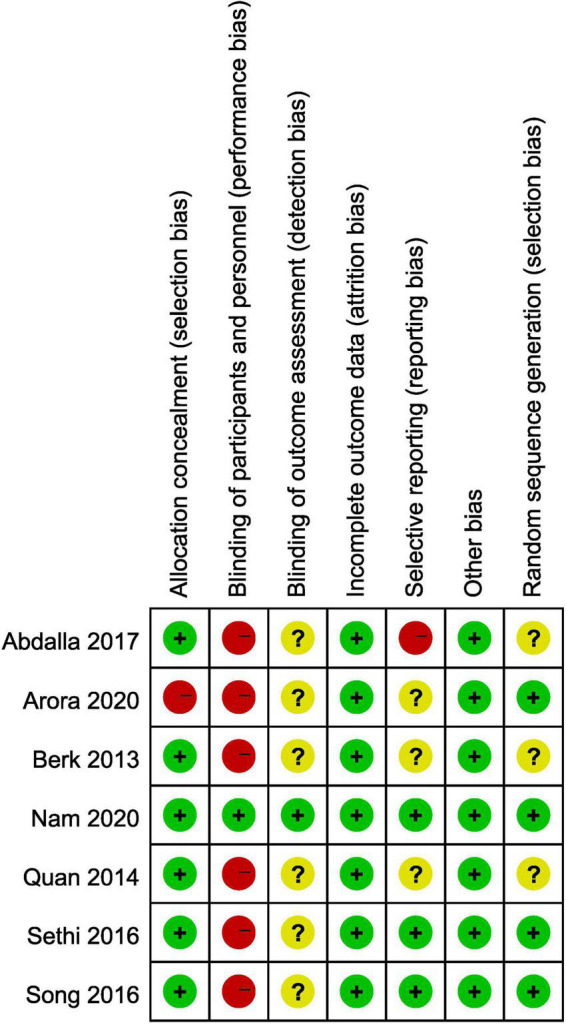
Summary of risk of bias.

All studies were assessed for risk of bias and methodological quality using the Cochrane Collaboration tool for risk of bias (Table 2). Details of the risk of bias are provided in Figure 2. The level of evidence quality for the outcomes is presented in the Supplementary Table 6.

**TABLE 2:** Assessment of risk of bias and methodological quality of the included studies based on the Cochrane Collaboration’s tool.

Study	Sequence generation	Allocation Concealment	Blinding	Incomplete outcome data	Selective outcome reporting
Berk et al. (21)	Unclear	Low risk	High risk	Low risk	Unclear risk
Quan et al. (9)	Unclear	Low risk	High risk	Low risk	Unclear risk
Song et al. (16)	Low risk	Low risk	High risk	Low risk	Low risk
Sethi et al. (17)	Low risk	Low risk	High risk	Low risk	Low risk
Abdalla et al. (19)	Unclear	Low risk	High risk	Low risk	High risk
Arora et al. (20)	Low risk	High risk	High risk	Low risk	Unclear risk
Nam et al. (10)	Low risk	Low risk	High risk	Low risk	Low risk

The summary of the meta-analyses is presented in the Supplementary Table 6.

### Primary outcome

#### Rate of cannulation success on the first attempt

**FIGURE 3:**
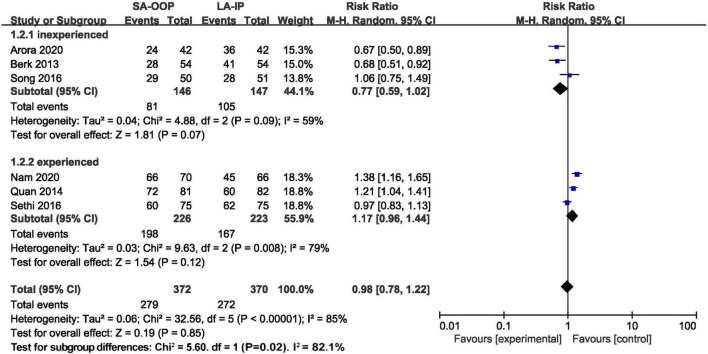
Forest plot depicting first-attempt success (subgroup analysis of special short axis technique).

**FIGURE 4:**
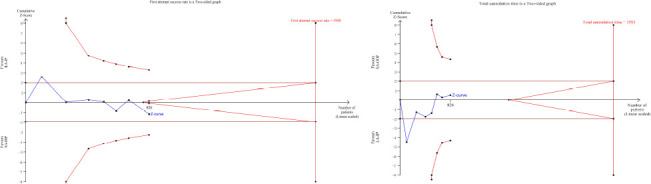
TSA results.

All studies included in this review reported the first-attempt success rates of both SA-OOP and LA-IP ultrasound-guided radial arterial cannulation. Compared with the SA-OOP group, the LA-IP group exhibited a higher first-attempt success rate (SA-OOP: 72.46%; LA-IP: 68.69%), but there was not statistically significant (RR = 1.03; 95% CI: 0.83 to 1.28; *P* = 0.79; *I*^2^ = 83.0%; Figure 3). TSA could not confirm this result, as the cumulative Z-curve did not cross the futility boundary. The TSA-adjusted 95% CI was 0.88 to 1.64 (Figure 4). However, statistically significant heterogeneity was noted among the included studies. Sensitivity analysis of the first-attempt success rate revealed no change in the seven studies (Supplementary Figure 7). Another sensitivity analysis indicated that there was not statistically significant between the modified SA-OOP, dynamic needle tip positioning, and LA-IP techniques (RR = 1.26; 95% CI: 0.96 to 1.64; *P* = 0.10; *I*^2^ = 52%; Figure 3). Meta-regression analysis indicated that the anaesthetic status of the patients and local anaesthesia before cannulation did not significantly impact the incidence of first-attempt success (*P* = 0.944 and *P* = 0.748, respectively).

Subgroup analysis was performed according to the operators’ experience (< 200 or ≥ 200 arterial cannulations). In the subgroup with less than 200 arterial cannulations, no significant difference in first attempt success rate was observed between the SA-OOP and LA-IP groups (RR = 0.77; 95% CI: 0.59 to 1.02; *P* = 0.07; *I*^2^ = 59.0%; Supplementary Figure 8). Similarly, in the subgroup with more than 200 arterial cannulations, no significant difference in primary outcome was observed between the SA-OOP and LA-IP groups (RR = 0.77; 95% CI: 0.59 to 1.02; *P* = 0.07; *I*^2^ = 59.0%; see Supplementary Figure 8). The GRADE quality of evidence was low.

### Secondary outcomes

#### Total time to successful cannulation

All seven studies (two high-quality and five low-quality) reported total time to successful cannulation. There was no significant difference in total time to successful cannulation between the SA-OOP and LA-IP groups (MD = –3.9; 95% CI:-18.30 to 10.49; *P* = 0.6; *I*^2^ = 97%). The cumulative Z-curve did not cross the futility boundary (Supplementary Figure 9).

Subgroup analysis was performed due to the statistical heterogeneity. The trend revealed that the final success rate was lower in the inexperienced subgroup by using the SA-OOP technique (RR = 0.938; 95% CI: 0.857 to 1.027; *P* = 0; *I*^2^ = 0; Supplementary Figure 9). Sensitivity analysis revealed that the study by Abdalla and colleagues was significantly different from other studies in terms of the total time to successful cannulation, which might contribute to heterogeneity. No publication bias was observed for this end point. Moderate-level evidence was observed regarding the total time to successful cannulation.

#### Number of cannulation attempts

Moderate-level evidence revealed that there was no difference in the number of cannulation attempts between the SA-OOP and LA-IP groups (MD = –0.01; 95% CI:–0.30 to 0.27; *P* = 0.93; *I*^2^ = 89%; Supplementary Figure 9). Subgroup analysis indicates that the number of punctures was significantly higher in inexperienced operators by using SA-OOP technique (Supplementary Figure 8).

#### Final success rate

Moderate –level evidence was observed regarding the final success of radial artery cannulation. All seven studies reported the final success rate of cannulation, two of which were unavailable due to double-arm zero events. There was no significant difference in final cannulation success rate between the SA-OOP and LA-IP groups (RR = 0.99; 95% CI: 0.96–1.02; *P* = 0.42; *I*^2^ = 0%; Table 2), with low heterogeneity among the studies (*I*^2^ = 0%). Egger’s test demonstrated that the publication bias for this outcome was significant (*P* = 0.037).

#### Ultrasonic location time

Moderate-quality evidence from four studies demonstrated that less ultrasonic location time was needed in the SA-OOP group than in the LA-IP group (MD = –13.67; 95% CI:-18.70 to –8.63; *P* = 0; *I*^2^ = 97%; Table 2). More ultrasonic location time was needed in the inexperienced subgroup (MD = –30.50; 95% CI:-42.31 to –18.69; *P* = 0; *I*^2^ = 0; see Supplementary Figure 9) using the SA-OOP technique. There was no publication bias in ultrasound location time according to the funnel plot and results of the Egger’s test (*P* = 0.141) and Begg’s test (*P* = 1).

#### Complications

Five complications of radial artery cannulation were included in this review; thrombosis and oedema could only be extracted from one study due to missing data and double-arm-zero events. There were no significant differences with regard to other complications, posterior arterial wall damage, vasospasm, and haematoma between the SA-OOP and LA-IP groups (Supplementary Figure 8).

## Discussion

### Main findings

This is the first systematic review and meta-analysis to compare the efficacy and safety of SA-OOP with LA-IP in ultrasound-guided radial arterial cannulation. The key findings of this meta-analysis are as follows. There were no significant differences in the first-attempt success rate, total time to successful cannulation, final success rate, and the incidence of complications between the LA-IP and SA-OOP groups. Ultrasonic location time was significantly lower in the SA-OOP group than in the LA-IP group.

### Comparisons with other meta-analyses

The main findings of this systematic review are consistent with previous reviews, including first-attempt success rate, and cannulation times. However, there are also several differences between this study and previous systematic reviews (8, 18). First, previous systematic reviews included different types of vessels such as the radial artery, femoral artery, subclavian vein, and internal jugular vein. Our systematic review included the most comprehensive trials comparing SA-OOP with LA-IP for ultrasound-guided radial arterial cannulation. Second, we performed TSA to provide conclusive evidence of the outcomes, the first-attempt success rate and total cannulation time, between the two approaches. However, the required information size of the two outcomes was not reached based on TSA. Third, previous systematic review and meta-analysis did not pay attention to the modified SA-OOP and dynamic needle tip positioning techniques. Our study performed subgroup analysis to investigate the effect of modified SA-OOP on the first-attempt success rate and total cannulation time. Fourthly, we evaluated the quality of evidence for outcomes using GRADE.

### Implications for clinical practice

Different clinical settings of the included studies may be a source of clinical heterogeneity. Given the increasing use of ultrasound in clinical settings, it is essential to identify the role of different approaches in ultrasound guidance for radial artery catheterisation. However, sensitivity analysis showed there was no significant difference in the first-attempt success rate between two approaches in different clinical settings. The sensitivity analysis showed that as for total cannulation time, the study from Abdalla and colleagues (19) provided a large source of heterogeneity, which decreased heterogeneity from 97 to 83% in this endpoint. Sensitivity analysis in hematoma showed great differences between the studies from Abdalla, (19) Nam, (10) Arora, (20), and Berk (21); however, we could not find the relationship among them.

Moreover, different techniques of ultrasound-guided arterial cannulation were used for the SA-OOP approach among studies. Compared with the conventional SA-OOP technique, modified SA-OOP and dynamic needle tip positioning significantly increased the success rate of first-attempt cannulation and total cannulation. Subgroup analysis indicated modified SA-OOP and dynamic needle tip positioning significantly increase first-attempt success rate, although no statistically significant test of interaction between subgroups was evident. The current evidence is still not sufficient to draw the conclusion that which approach is more efficient. Therefore, further trials are warranted.

The modified SA-OOP technique might hold an advantage in the rate of cannulation success on the first attempt, although no statistically significant results were obtained in our research. Therefore, we speculate that especially in the patients with difficulties, the special type of SA-OOP technology may improve the first puncture success rate.

Furthermore, operator experience is a source of clinical heterogeneity. Subgroup analysis revealed that inexperienced operators using the SA-OOP approach may result in a higher rate of post-wall damage, haematoma and increased ultrasonic location time.

Certain guidelines (6) recommend ultrasound guidance in special types of people, such as children, low blood volume, low blood pressure, low cardiac output, no or almost no arterial pulse, arterial spasm or hematoma, and radial artery puncture with long limbs, but it is still unclear which technique is more suitable for these types of people.

### Strengths and limitations

A major strength of this meta-analysis was the compliance with the PRISMA guidelines and the recommendations of the Cochrane Collaboration. Our study was also registered with protocol in PROSPERO. In order to increase the robustness of this meta-analysis, we applied TSA to assess the impact of random error and repetitive testing.

This study also had several limitations. First, double-blinding was not performed in most of the included studies due to the trial features, which may have resulted in performance and detection bias. Thus, two investigators who were unaware of the group independently extracted data to decrease performance and detection bias. Second, although subgroup analyses and meta-regression were performed with regard to different experience levels of the staff, type of SA-OOP technique, the anaesthetic status of the patients and local anaesthesia before cannulation, there was considerable clinical and statistical heterogeneity among the included studies.

## Conclusion

In conclusion, there were no significant differences in the success rate of the first attempt and total cannulation time between SA-OOP and LA-IP. However, the results should be interpreted with caution due to the significant heterogeneity among studies. Our findings highlight the potential of the SA-OOP technique, but further well-designed robust randomised controlled trials are warranted to investigate this technique in patients undergoing ultrasound-guided radial artery cannulation.

## Data availability statement

The original contributions presented in this study are included in the article/Supplementary material, further inquiries can be directed to the corresponding author.

## Author contributions

ML and Y-LW designed the study. W-YD, X-XS, and HZ selected the studies. W-YD, X-XS, and YL extracted the data. ML, W-YD, X-XS, and YL performed the analyses. X-XS and W-YD interpreted the data and drafted the manuscript. All authors approved the version to be published.

## References

[1] White L Halpin A Turner M Wallace L. Ultrasound-guided radial artery cannulation in adult and paediatric populations: a systematic review and meta-analysis. *Br J Anaesth.* (2016) 116:610–7. 10.1093/bja/aew097 27106964

[2] Gu WJ Wu XD Wang F Ma ZL Gu XP. Ultrasound guidance facilitates radial artery catheterization: a meta-analysis with trial sequential analysis of randomized controlled trials. *Chest.* (2016) 149:166–79. 10.1378/chest.15-1784 26426094

[3] Ueda K Puangsuvan S Hove MA Bayman EO. Ultrasound visual image-guided vs Doppler auditory-assisted radial artery cannulation in infants and small children by non-expert anaesthesiologists: a randomized prospective study. *Br J Anaesth.* (2013) 110:281–6. 10.1093/bja/aes383 23151422

[4] Latham GJ Veneracion ML Joffe DC Bosenberg AT Flack SH Low DK. High-frequency micro-ultrasound for vascular access in young children–a feasibility study by the high-frequency ultrasound in kids study (HUSKY) group. *Paediatr Anaesth.* (2013) 23:529–35. 10.1111/pan.12131 23445349

[5] Brzezinski M Luisetti T London MJ. Radial artery cannulation: a comprehensive review of recent anatomic and physiologic investigations. *Anesth Analg.* (2009) 109:1763–81. 10.1213/ANE.0b013e3181bbd416 19923502

[6] Troianos CA Hartman GS Glas KE Skubas NJ Eberhardt RT Walker JD Special articles: guidelines for performing ultrasound guided vascular cannulation: recommendations of the American society of echocardiography and the society of cardiovascular anesthesiologists. *Anesth Analg.* (2012) 114:46–72. 10.1213/ANE.0b013e3182407cd8 22127816

[7] Lamperti M Biasucci DG Disma N Pittiruti M Breschan C Vailati D European society of anaesthesiology guidelines on peri-operative use of ultrasound-guided for vascular access (PERSEUS vascular access). *Eur J Anaesthesiol.* (2020) 37:344–76. 32265391 10.1097/EJA.0000000000001180

[8] Liu C Mao Z Kang H Hu X Jiang S Hu P Comparison between the long-axis/in-plane and short-axis/out-of-plane approaches for ultrasound-guided vascular catheterization: an updated meta-analysis and trial sequential analysis. *Ther Clin Risk Manag.* (2018) 14:331–40. 10.2147/TCRM.S152908 29503552 PMC5824754

[9] Quan Z Tian M Chi P Cao Y Li X Peng K. Modified short-axis out-of-plane ultrasound versus conventional long-axis in-plane ultrasound to guide radial artery cannulation: a randomized controlled trial. *Anesth Analg.* (2014) 119:163–9. 10.1213/ANE.0000000000000242 24806143

[10] Nam K Jeon Y Yoon S Kwon SM Kang P Cho YJ Ultrasound-guided radial artery cannulation using dynamic needle tip positioning versus conventional long-axis in-plane techniques in cardiac surgery patients: a randomized, controlled trial. *Minerva Anestesiol.* (2020) 86:30–7. 10.23736/S0375-9393.19.13646-2 31213045

[11] Moher D Liberati A Tetzlaff J Altman DG. Preferred reporting items for systematic reviews and meta-analyses: the PRISMA statement. *BMJ.* (2009) 339:b2535. 10.1136/bmj.b2535 PMC2714657 19622551

[12] Higgins JPT Thomas J Chandler J Cumpston M Li T Page MJ editors. *Cochrane Handbook for Systematic Reviews of Interventions Version 6.3.* (2022). Available online at: www.training.cochrane.org/handbook (accessed February 2022).

[13] Xu C Zhou X Zorzela L Ju K Furuya-Kanamori L Lin L Utilization of the evidence from studies with no events in meta-analyses of adverse events: an empirical investigation. *BMC Med.* (2021) 19:141. 10.1186/s12916-021-02008-2 34126999 PMC8204528

[14] Luo D Wan X Liu J Tong T. Optimally estimating the sample mean from the sample size, median, mid-range, and/or mid-quartile range. *Stat Methods Med Res.* (2018) 27:1785–805. 27683581 10.1177/0962280216669183

[15] Wan X Wang W Liu J Tong T. Estimating the sample mean and standard deviation from the sample size, median, range and/or interquartile range. *BMC Med Res Methodol.* (2014) 14:135. 10.1186/1471-2288-14-135 25524443 PMC4383202

[16] Song IK Choi JY Lee JH Kim EH Kim HJ Kim HS Short-axis/out-of-plane or long-axis/in-plane ultrasound-guided arterial cannulation in children: a randomised controlled trial. *Eur J Anaesthesiol.* (2016) 33:522–7. 26986774 10.1097/EJA.0000000000000453

[17] Sethi S Maitra S Saini V Samra T Malhotra SK. Comparison of short-axis out-of-plane versus long-axis in-plane ultrasound-guided radial arterial cannulation in adult patients: a randomized controlled trial. *J Anesth.* (2017) 31:89–94. 10.1007/s00540-016-2270-6 27761661

[18] Gao YB Yan JH Ma JM Liu XN Dong JY Sun F Effects of long axis in-plane vs short axis out-of-plane techniques during ultrasound-guided vascular access. *Am J Emerg Med.* (2016) 34:778–83. 26830218 10.1016/j.ajem.2015.12.092

[19] Abdalla UE Elmaadawey A Kandeel A. Oblique approach for ultrasound-guided radial artery catheterization vs transverse and longitudinal approaches, a randomized trial. *J Clin Anesth.* (2017) 36:98–101. 10.1016/j.jclinane.2016.10.016 28183585

[20] Arora NR Maddali MM Al-Sheheimi RAR Al-Mughairi H Panchatcharam SM. Ultrasound-Guided out-of-plane versus in-plane radial artery cannulation in adult cardiac surgical patients. *J Cardiothorac Vasc Anesth.* (2021) 35:84–8. 10.1053/j.jvca.2020.08.025 32891521

[21] Berk D Gurkan Y Kus A Ulugol H Solak M Toker K. Ultrasound-guided radial arterial cannulation: long axis/in-plane versus short axis/out-of-plane approaches? *J Clin Monitor Comput.* (2013) 27:319–24. 10.1007/s10877-013-9437-6 23417581

