# Corrigendum: Comparison of out-of-plane short axis with in-plane long axis for ultrasound-guided radial arterial cannulation: A systematic review with trial sequential analysis of randomised controlled trials

**DOI:** 10.3389/fcvm.2023.1191088

**Published:** 2023-04-04

**Authors:** Xia-xuan Sun, Meng Lv, Wen-ya Du, Yi Liu, Haixia Zhang, Yue-lan Wang

**Affiliations:** ^1^ Department of Anesthesiology, The First Affiliated Hospital of Shandong First Medical University & Shandong Provincial Qianfoshan Hospital, Shandong Institute of Anesthesia and Respiratory Critical Care Medicine, Jinan, China; ^2^ Shandong First Medical University & Shandong Academy of Medical Sciences, Jinan, China

**Keywords:** cannulation, catheterisation, long-axis in-plane, radial artery, short-axis out-ofplane, ultrasound guidance

A Corrigendum on Comparison of out-of-plane short axis with in-plane long axis for ultrasound-guided radial arterial cannulation: A systematic review with trial sequential analysis of randomised controlled trials Sun XX, Lv M, Du WY, Liu Y, Zhang H and Wang YL. (2022) Front. Cardiovasc. Med. 9:983532. doi: 10.3389/fcvm.2022.983532

In the published article, there was an error in affiliation 1. Instead of “Graduate School, Shandong First Medical University & Shandong Academy of Medical Sciences, Jinan, China” it should be “Department of Anesthesiology, The First Affiliated Hospital of Shandong First Medical University & Shandong Provincial Qianfoshan Hospital, Shandong Institute of Anesthesia and Respiratory Critical Care Medicine, Jinan, China”.

In the published article, there was an error in affiliations 2. Instead of “Department of Anesthesiology, The First Affiliated Hospital of Shandong First Medical University & Shandong Provincial Qianfoshan Hospital, Jinan, China”, it should be “Shandong First Medical University & Shandong Academy of Medical Sciences, Jinan, China”.

In the published article, there was an error regarding the affiliations for Meng Lv. As well as having affiliations 2, they should also have affiliation 1 ‘Department of Anesthesiology, The First Affiliated Hospital of Shandong First Medical University & Shandong Provincial Qianfoshan Hospital, Shandong Institute of Anesthesia and Respiratory Critical Care Medicine, Jinan, China’.

In the published article, there was an error regarding the affiliation for Yi Liu and Haixia Zhang. Their affiliation was displayed as 2 “Department of Anesthesiology, The First Affiliated Hospital of Shandong First Medical University & Shandong Provincial Qianfoshan Hospital, Jinan, China”. The correct affiliation is 1 “Department of Anesthesiology, The First Affiliated Hospital of Shandong First Medical University & Shandong Provincial Qianfoshan Hospital, Shandong Institute of Anesthesia and Respiratory Critical Care Medicine, Jinan, China”.

The authors apologize for these errors and state that this does not change the scientific conclusions of the article in any way. The original article has been updated.

