# Supplementary material for: Comparison of out-of-plane short axis with in-plane long axis for ultrasound-guided radial arterial cannulation: A systematic review with trial sequential analysis of randomised controlled trials
Source: Front Cardiovasc Med. 2022 Oct 12;9:983532. doi: 10.3389/fcvm.2022.983532 (PMC9596768; doi:10.3389/fcvm.2022.983532)

Supplementary Material

**Supplement 1.** Databases search strategies.

**Supplement 2.** Risk of bias assessment for included studies.

**Supplement 3.** The GRADE evidence profile for ultrasound-guided radial arterial cannulation.

**Supplement 4.** Sensitivity analysis

**Supplement 5.** Begg’s and Egger’s tests for the primary outcome

**Supplement 6.** Summary of the meta-analyses

**Supplement 7.** The sensitivity analysis of primary outcome

**Supplement 8.** Subgroup analysis based on operation experience –SA-OOP vs LA-IP

**Supplement 9.** Forrest and funnel plots for secondary outcomes

**Supplement 10.** Forrest and funnel plots for primary outcomes

This supplementary material has been provided by the authors to give readers additional information about their work.

**Supplement 1.** Databases search strategies

PubMed

#1 "Radial Artery"[MeSH Terms]

#2 "radial artery"[Title/Abstract] OR "arteries radial"[Title/Abstract] OR "artery radial"[Title/Abstract] OR "radial arteries"[Title/Abstract]

#3 "Catheterization"[MeSH Terms]

#4 "Catheterization"[Title/Abstract] OR "Catheterizations"[Title/Abstract] OR "Cannulation"[Title/Abstract] OR "Cannulations"[Title/Abstract]

#5 "catheterization, peripheral"[MeSH Terms]

#6 "catheterization peripheral"[Title/Abstract] OR "peripheral catheterization"[Title/Abstract] OR (("catheterisation"[All Fields] OR "Catheterization"[MeSH Terms] OR "Catheterization"[All Fields] OR "catheterisations"[All Fields] OR "catheterising"[All Fields] OR "catheterism"[All Fields] OR "catheterisms"[All Fields] OR "catheterise"[All Fields] OR "catheterised"[All Fields] OR "Catheterizations"[All Fields] OR "catheterize"[All Fields] OR "catheterized"[All Fields] OR "catheterizing"[All Fields]) AND "Peripheral"[Title/Abstract]) OR "peripheral catheterizations"[Title/Abstract] OR (("catheterisation"[All Fields] OR "Catheterization"[MeSH Terms] OR "Catheterization"[All Fields] OR "catheterisations"[All Fields] OR "catheterising"[All Fields] OR "catheterism"[All Fields] OR "catheterisms"[All Fields] OR "catheterise"[All Fields] OR "catheterised"[All Fields] OR "Catheterizations"[All Fields] OR "catheterize"[All Fields] OR "catheterized"[All Fields] OR "catheterizing"[All Fields]) AND "peripheral arterial"[Title/Abstract]) OR "peripheral arterial catheterization"[Title/Abstract] OR ((("arterialization"[All Fields] OR "arterializations"[All Fields] OR "arterialize"[All Fields] OR "arterialized"[All Fields] OR "arterializing"[All Fields] OR "arterially"[All Fields] OR "arterials"[All Fields] OR "arterie"[All Fields] OR "arteries"[MeSH Terms] OR "arteries"[All Fields] OR "Arterial"[All Fields] OR "arteris"[All Fields] OR "artery"[All Fields] OR "arterious"[All Fields] OR "artery s"[All Fields] OR "arterys"[All Fields]) AND ("catheterisation"[All Fields] OR "Catheterization"[MeSH Terms] OR "Catheterization"[All Fields] OR "catheterisations"[All Fields] OR "catheterising"[All Fields] OR "catheterism"[All Fields] OR "catheterisms"[All Fields] OR "catheterise"[All Fields] OR "catheterised"[All Fields] OR "Catheterizations"[All Fields] OR "catheterize"[All Fields] OR "catheterized"[All Fields] OR "catheterizing"[All Fields])) AND "Peripheral"[Title/Abstract]) OR (("catheterisation"[All Fields] OR "Catheterization"[MeSH Terms] OR "Catheterization"[All Fields] OR "catheterisations"[All Fields] OR "catheterising"[All Fields] OR "catheterism"[All Fields] OR "catheterisms"[All Fields] OR "catheterise"[All Fields] OR "catheterised"[All Fields] OR "Catheterizations"[All Fields] OR "catheterize"[All Fields] OR "catheterized"[All Fields] OR "catheterizing"[All Fields]) AND "peripheral arterial"[Title/Abstract]) OR (("Peripheral"[All Fields] OR "peripherally"[All Fields] OR "peripherals"[All Fields] OR "periphereal"[All Fields] OR "peripheric"[All Fields] OR "peripherically"[All Fields]) AND "arterial catheterizations"[Title/Abstract]) OR "arterial catheterization peripheral"[Title/Abstract]

#7 "Catheters"[MeSH Terms]

#8 "Catheters"[Title/Abstract] OR "Catheter"[Title/Abstract]

#9 "Vascular Access Devices"[MeSH Terms]

#10 "vascular access devices"[Title/Abstract] OR "device vascular access"[Title/Abstract] OR (("device s"[All Fields] OR "equipment and supplies"[MeSH Terms] OR ("equipment"[All Fields] AND "supplies"[All Fields]) OR "equipment and supplies"[All Fields] OR "Device"[All Fields] OR "instrumentation"[MeSH Subheading] OR "instrumentation"[All Fields] OR "Devices"[All Fields]) AND "vascular access"[Title/Abstract]) OR "vascular access device"[Title/Abstract] OR "venous reservoirs"[Title/Abstract] OR "reservoir venous"[Title/Abstract] OR (("Reservoir"[All Fields] OR "reservoir s"[All Fields] OR "Reservoirs"[All Fields]) AND "Venous"[Title/Abstract]) OR "venous reservoir"[Title/Abstract] OR "vascular access ports"[Title/Abstract] OR "vascular catheters"[Title/Abstract] OR "catheter vascular"[Title/Abstract] OR "catheters vascular"[Title/Abstract] OR "vascular catheter"[Title/Abstract] OR "intra arterial lines"[Title/Abstract] OR "intra arterial lines"[Title/Abstract] OR "intra arterial line"[Title/Abstract] OR "line intra arterial"[Title/Abstract] OR (("line s"[All Fields] OR "long interspersed nucleotide elements"[MeSH Terms] OR ("long"[All Fields] AND "interspersed"[All Fields] AND "nucleotide"[All Fields] AND "elements"[All Fields]) OR "long interspersed nucleotide elements"[All Fields] OR "Lines"[All Fields]) AND "Intra-Arterial"[Title/Abstract]) OR "arterial lines"[Title/Abstract] OR "arterial line"[Title/Abstract] OR "line arterial"[Title/Abstract] OR "lines arterial"[Title/Abstract]

#11 "Punctures"[MeSH Terms]

#12 "Punctures"[Title/Abstract] OR "Puncture"[Title/Abstract] OR "Micropunctures"[Title/Abstract] OR "Micropuncture"[Title/Abstract]

#13 "Ultrasonography"[MeSH Terms]

#14 "diagnostic imaging"[MeSH Subheading] OR ("diagnostic"[All Fields] AND "imaging"[All Fields]) OR "diagnostic imaging"[All Fields] OR "ultrasonography"[All Fields] OR "ultrasonography"[MeSH Terms] OR "ultrasonographies"[All Fields] OR ("ultrasonography"[MeSH Terms] OR "ultrasonography"[All Fields] OR ("diagnostic"[All Fields] AND "ultrasound"[All Fields]) OR "diagnostic ultrasound"[All Fields]) OR ("ultrasonography"[MeSH Terms] OR "ultrasonography"[All Fields] OR ("diagnostic"[All Fields] AND "ultrasounds"[All Fields]) OR "diagnostic ultrasounds"[All Fields]) OR ("ultrasonography"[MeSH Terms] OR "ultrasonography"[All Fields] OR ("ultrasound"[All Fields] AND "diagnostic"[All Fields]) OR "ultrasound diagnostic"[All Fields]) OR ("ultrasonography"[MeSH Terms] OR "ultrasonography"[All Fields] OR ("ultrasounds"[All Fields] AND "diagnostic"[All Fields]) OR "ultrasounds diagnostic"[All Fields]) OR ("ultrasonography"[MeSH Terms] OR "ultrasonography"[All Fields] OR ("ultrasound"[All Fields] AND "imaging"[All Fields]) OR "ultrasound imaging"[All Fields]) OR ("ultrasonography"[MeSH Terms] OR "ultrasonography"[All Fields] OR ("imaging"[All Fields] AND "ultrasound"[All Fields]) OR "imaging ultrasound"[All Fields]) OR ("ultrasonography"[MeSH Terms] OR "ultrasonography"[All Fields] OR ("imagings"[All Fields] AND "ultrasound"[All Fields])) OR ("diagnostic imaging"[MeSH Subheading] OR ("diagnostic"[All Fields] AND "imaging"[All Fields]) OR "diagnostic imaging"[All Fields] OR "echotomography"[All Fields] OR "ultrasonography"[MeSH Terms] OR "ultrasonography"[All Fields]) OR ("ultrasonography"[MeSH Terms] OR "ultrasonography"[All Fields] OR ("ultrasonic"[All Fields] AND "imaging"[All Fields]) OR "ultrasonic imaging"[All Fields]) OR ("ultrasonography"[MeSH Terms] OR "ultrasonography"[All Fields] OR ("imaging"[All Fields] AND "ultrasonic"[All Fields]) OR "imaging ultrasonic"[All Fields]) OR ("ultrasonography"[MeSH Terms] OR "ultrasonography"[All Fields] OR ("sonography"[All Fields] AND "medical"[All Fields]) OR "sonography medical"[All Fields]) OR ("ultrasonography"[MeSH Terms] OR "ultrasonography"[All Fields] OR ("medical"[All Fields] AND "sonography"[All Fields]) OR "medical sonography"[All Fields]) OR ("ultrasonography"[MeSH Terms] OR "ultrasonography"[All Fields] OR ("ultrasonographic"[All Fields] AND "imaging"[All Fields]) OR "ultrasonographic imaging"[All Fields]) OR ("ultrasonography"[MeSH Terms] OR "ultrasonography"[All Fields] OR ("imaging"[All Fields] AND "ultrasonographic"[All Fields]) OR "imaging ultrasonographic"[All Fields]) OR ("ultrasonography"[MeSH Terms] OR "ultrasonography"[All Fields] OR ("imagings"[All Fields] AND "ultrasonographic"[All Fields])) OR ("ultrasonography"[MeSH Terms] OR "ultrasonography"[All Fields] OR ("ultrasonographic"[All Fields] AND "imagings"[All Fields]) OR "ultrasonographic imagings"[All Fields]) OR ("diagnostic imaging"[MeSH Subheading] OR ("diagnostic"[All Fields] AND "imaging"[All Fields]) OR "diagnostic imaging"[All Fields] OR "echography"[All Fields] OR "ultrasonography"[MeSH Terms] OR "ultrasonography"[All Fields] OR "echographies"[All Fields]) OR ("ultrasonography"[MeSH Terms] OR "ultrasonography"[All Fields] OR ("diagnosis"[All Fields] AND "ultrasonic"[All Fields]) OR "diagnosis ultrasonic"[All Fields]) OR ("ultrasonography"[MeSH Terms] OR "ultrasonography"[All Fields] OR ("diagnoses"[All Fields] AND "ultrasonic"[All Fields]) OR "diagnoses ultrasonic"[All Fields]) OR ("ultrasonography"[MeSH Terms] OR "ultrasonography"[All Fields] OR ("ultrasonic"[All Fields] AND "diagnoses"[All Fields]) OR "ultrasonic diagnoses"[All Fields]) OR ("diagnostic imaging"[MeSH Subheading] OR ("diagnostic"[All Fields] AND "imaging"[All Fields]) OR "diagnostic imaging"[All Fields] OR ("ultrasonic"[All Fields] AND "diagnosis"[All Fields]) OR "ultrasonic diagnosis"[All Fields] OR "ultrasonography"[MeSH Terms] OR "ultrasonography"[All Fields] OR ("ultrasonic"[All Fields] AND "diagnosis"[All Fields])) OR ("ultrasonography"[MeSH Terms] OR "ultrasonography"[All Fields] OR ("echotomography"[All Fields] AND "computer"[All Fields]) OR "echotomography computer"[All Fields]) OR ("ultrasonography"[MeSH Terms] OR "ultrasonography"[All Fields] OR ("computer"[All Fields] AND "echotomography"[All Fields]) OR "computer echotomography"[All Fields]) OR ("ultrasonography"[MeSH Terms] OR "ultrasonography"[All Fields] OR ("tomography"[All Fields] AND "ultrasonic"[All Fields]) OR "tomography ultrasonic"[All Fields])

#15 "Ultrasonics"[MeSH Terms]

#16 "Ultrasonics"[Title/Abstract] OR "Ultrasonic"[Title/Abstract]

#17 "Ultrasonic Waves"[MeSH Terms]

#18 (("ultrasonic waves"[Title/Abstract] OR "ultrasonic wave"[Title/Abstract] OR "wave ultrasonic"[Title/Abstract] OR "waves ultrasonic"[Title/Abstract] OR "ultrasonic vibration"[Title/Abstract] OR "ultrasonic vibrations"[Title/Abstract] OR "vibration ultrasonic"[Title/Abstract] OR (("vibrate"[All Fields] OR "vibrated"[All Fields] OR "vibrates"[All Fields] OR "vibrating"[All Fields] OR "Vibration"[MeSH Terms] OR "Vibration"[All Fields] OR "Vibrations"[All Fields] OR "vibrational"[All Fields] OR "vibrator"[All Fields] OR "vibrators"[All Fields]) AND "Ultrasonic"[Title/Abstract]) OR "ultrasound radiation"[Title/Abstract] OR "radiation ultrasound"[Title/Abstract] OR "ultrasound waves"[Title/Abstract] OR "ultrasound wave"[Title/Abstract] OR "wave ultrasound"[Title/Abstract] OR "waves ultrasound"[Title/Abstract] OR "pulsed ultrasound"[Title/Abstract] OR "pulsed ultrasounds"[Title/Abstract] OR "ultrasound pulsed"[Title/Abstract] OR "ultrasounds pulsed"[Title/Abstract] OR "low intensity pulsed ultrasound"[Title/Abstract]) AND "OR"[Title/Abstract]) OR "low intensity pulsed ultrasound"[Title/Abstract] OR "low intensity pulsed ultrasound"[Title/Abstract] OR "low intensity pulsed ultrasounds"[Title/Abstract] OR (("pulse"[MeSH Terms] OR "pulse"[All Fields] OR "heart rate"[MeSH Terms] OR ("heart"[All Fields] AND "rate"[All Fields]) OR "heart rate"[All Fields] OR "pulses"[All Fields] OR "pulse s"[All Fields] OR "Pulsed"[All Fields] OR "pulsing"[All Fields]) AND "ultrasound low intensity"[Title/Abstract]) OR (("ultrasonic waves"[MeSH Terms] OR ("Ultrasonic"[All Fields] AND "Waves"[All Fields]) OR "ultrasonic waves"[All Fields] OR ("Pulsed"[All Fields] AND "Ultrasounds"[All Fields]) OR "pulsed ultrasounds"[All Fields]) AND "Low-Intensity"[Title/Abstract]) OR "ultrasound low intensity pulsed"[Title/Abstract] OR (("diagnostic imaging"[MeSH Subheading] OR ("diagnostic"[All Fields] AND "imaging"[All Fields]) OR "diagnostic imaging"[All Fields] OR "Ultrasound"[All Fields] OR "ultrasonography"[MeSH Terms] OR "ultrasonography"[All Fields] OR "ultrasonics"[MeSH Terms] OR "ultrasonics"[All Fields] OR "Ultrasounds"[All Fields] OR "ultrasound s"[All Fields]) AND "low intensity pulsed"[Title/Abstract]) OR (("Low"[All Fields] AND ("intense"[All Fields] OR "intensely"[All Fields] OR "intensities"[All Fields] OR "Intensity"[All Fields] OR "intensively"[All Fields]) AND ("pulse"[MeSH Terms] OR "pulse"[All Fields] OR "heart rate"[MeSH Terms] OR ("heart"[All Fields] AND "rate"[All Fields]) OR "heart rate"[All Fields] OR "pulses"[All Fields] OR "pulse s"[All Fields] OR "Pulsed"[All Fields] OR "pulsing"[All Fields])) AND "ultrasound radiation"[Title/Abstract])

#19 "ultrasonography, doppler"[MeSH Terms]

#20 "ultrasonography doppler"[Title/Abstract] OR "doppler ultrasound"[Title/Abstract] OR "doppler ultrasounds"[Title/Abstract] OR "ultrasound doppler"[Title/Abstract] OR "ultrasounds doppler"[Title/Abstract] OR "doppler ultrasonography"[Title/Abstract] OR "doppler ultrasound imaging"[Title/Abstract] OR (("Doppler"[All Fields] OR "doppler s"[All Fields] OR "dopplers"[All Fields]) AND "ultrasound imagings"[Title/Abstract]) OR "imaging doppler ultrasound"[Title/Abstract] OR (("image"[All Fields] OR "image s"[All Fields] OR "imaged"[All Fields] OR "imager"[All Fields] OR "imager s"[All Fields] OR "imagers"[All Fields] OR "images"[All Fields] OR "Imaging"[All Fields] OR "imaging s"[All Fields] OR "Imagings"[All Fields]) AND "doppler ultrasound"[Title/Abstract]) OR "ultrasound imaging doppler"[Title/Abstract] OR ((("diagnostic imaging"[MeSH Subheading] OR ("diagnostic"[All Fields] AND "Imaging"[All Fields]) OR "diagnostic imaging"[All Fields] OR "Ultrasound"[All Fields] OR "Ultrasonography"[MeSH Terms] OR "Ultrasonography"[All Fields] OR "ultrasonics"[MeSH Terms] OR "ultrasonics"[All Fields] OR "Ultrasounds"[All Fields] OR "ultrasound s"[All Fields]) AND ("image"[All Fields] OR "image s"[All Fields] OR "imaged"[All Fields] OR "imager"[All Fields] OR "imager s"[All Fields] OR "imagers"[All Fields] OR "images"[All Fields] OR "Imaging"[All Fields] OR "imaging s"[All Fields] OR "Imagings"[All Fields])) AND "Doppler"[Title/Abstract])

#21 #1 OR #2

#22 #3 OR #4 OR #5 OR #6 OR #7 OR #8 OR #9 OR #10 OR #11 OR #12

#23 #13 OR #14 OR #15 OR #16 OR #17 OR #18 OR #19 OR #20

#24 #21 AND #22 AND #23

#25 (randomized controlled trial[pt] OR controlled clinical trial[pt] OR randomized[tiab] OR placebo[tiab] OR clinical trials as topic[mesh:noexp] OR randomly[tiab] OR trial[ti]) NOT (animals [mh] NOT (humans [mh] AND animals[mh]))

#26 #24 AND #25

Cochrane

#1 MeSH descriptor: [Radial Artery] explode all trees

#2 (Radial Artery):ti,ab,kw OR (Arteries, Radial):ti,ab,kw OR (Artery, Radial):ti,ab,kw OR (Radial Arteries):ti,ab,kw

#3 MeSH descriptor: [Catheterization] explode all trees

#4 (Catheterization):ti,ab,kw OR (Catheterizations):ti,ab,kw OR (Cannulation):ti,ab,kw OR (Cannulations):ti,ab,kw

#5 MeSH descriptor: [Catheterization, Peripheral] explode all trees

#6 (Catheterization, Peripheral):ti,ab,kw OR (Peripheral Catheterization):ti,ab,kw OR (Catheterizations, Peripheral):ti,ab,kw OR (Peripheral Catheterizations):ti,ab,kw OR (Catheterization, Bronchial):ti,ab,kw OR (Bronchial Catheterization):ti,ab,kw OR (Bronchial Catheterizations):ti,ab,kw OR (Catheterizations, Bronchial):ti,ab,kw OR (Catheterization, Peripheral Arterial):ti,ab,kw OR (Peripheral Arterial Catheterization):ti,ab,kw OR (Arterial Catheterizations, Peripheral):ti,ab,kw OR (Catheterizations, Peripheral Arterial):ti,ab,kw OR (Peripheral Arterial Catheterizations):ti,ab,kw OR (Arterial Catheterization, Peripheral):ti,ab,kw OR (Peripheral Venous Catheterization):ti,ab,kw OR (Catheterizations, Peripheral Venous):ti,ab,kw OR (Peripheral Venous Catheterizations):ti,ab,kw OR (Venous Catheterizations, Peripheral):ti,ab,kw OR (Venous Catheterization, Peripheral):ti,ab,kw OR (LIPUS):ti,ab,kw OR (Catheterization, Peripheral Venous):ti,ab,kw

#7 MeSH descriptor: [Catheters] explode all trees

#8 (Catheters):ti,ab,kw OR(Catheter):ti,ab,kw

#9 MeSH descriptor: [Vascular Access Devices] explode all trees

#10 (Vascular Access Devices):ti,ab,kw OR(Device, Vascular Access):ti,ab,kw OR (Devices, Vascular Access):ti,ab,kw OR (Vascular Access Device):ti,ab,kw OR (Venous Reservoirs):ti,ab,kw OR (Reservoir, Venous):ti,ab,kw OR (Reservoirs, Venous):ti,ab,kw OR (Venous Reservoir):ti,ab,kw OR (Vascular Access Ports):ti,ab,kw OR (Vascular Catheters):ti,ab,kw OR (Catheter, Vascular):ti,ab,kw OR (Catheters, Vascular):ti,ab,kw OR (Vascular Catheter):ti,ab,kw OR (Intra-Arterial Lines):ti,ab,kw OR (Intra Arterial Lines):ti,ab,kw OR (Intra-Arterial Line):ti,ab,kw OR (Line, Intra-Arterial):ti,ab,kw OR (Lines, Intra-Arterial):ti,ab,kw OR (Arterial Lines):ti,ab,kw OR (Arterial Line):ti,ab,kw OR (Line, Arterial):ti,ab,kw OR (Lines, Arterial):ti,ab,kw

#11 MeSH descriptor: [Punctures] explode all trees

#12 (Punctures):ti,ab,kw OR (Puncture):ti,ab,kw OR (Micropunctures):ti,ab,kw OR (Micropuncture):ti,ab,kw

#13 MeSH descriptor: [Ultrasonography] explode all trees

#14 (Ultrasonography):ti,ab,kw OR (Diagnostic Ultrasound):ti,ab,kw OR (Diagnostic Ultrasounds):ti,ab,kw OR (Ultrasound, Diagnostic):ti,ab,kw OR (Ultrasounds, Diagnostic):ti,ab,kw OR (Ultrasound Imaging):ti,ab,kw OR (Imaging, Ultrasound):ti,ab,kw OR (Imagings, Ultrasound):ti,ab,kw OR (Echotomography):ti,ab,kw OR (Ultrasonic Imaging):ti,ab,kw OR (Imaging, Ultrasonic):ti,ab,kw OR (Sonography, Medical):ti,ab,kw OR (Medical Sonography):ti,ab,kw OR (Ultrasonographic Imaging):ti,ab,kw OR (Imaging, Ultrasonographic):ti,ab,kw OR (Imagings, Ultrasonographic):ti,ab,kw OR (Ultrasonographic Imagings):ti,ab,kw OR (Echography):ti,ab,kw OR (Diagnosis, Ultrasonic):ti,ab,kw OR (Diagnoses, Ultrasonic):ti,ab,kw OR (Ultrasonic Diagnoses):ti,ab,kw OR (Ultrasonic Diagnosis):ti,ab,kw OR (Echotomography, Computer):ti,ab,kw OR (Computer Echotomography):ti,ab,kw OR (Tomography, Ultrasonic):ti,ab,kw OR (Ultrasonic Tomography):ti,ab,kw

#15 MeSH descriptor: [Ultrasonics] explode all trees

#16 (Ultrasonics):ti,ab,kw OR (Ultrasonic):ti,ab,kw

#17 MeSH descriptor: [Ultrasonic Waves] explode all trees

#18 (Ultrasonic Waves):ti,ab,kw OR (Ultrasonic Wave):ti,ab,kw OR (Wave, Ultrasonic):ti,ab,kw OR (Waves, Ultrasonic):ti,ab,kw OR (Ultrasonic Vibration):ti,ab,kw OR (Ultrasonic Vibrations):ti,ab,kw OR (Vibration, Ultrasonic):ti,ab,kw OR (Vibrations, Ultrasonic):ti,ab,kw OR (Ultrasound Radiation):ti,ab,kw OR (Radiation, Ultrasound):ti,ab,kw OR (Ultrasound Waves):ti,ab,kw OR (Ultrasound Wave):ti,ab,kw OR (Wave, Ultrasound):ti,ab,kw OR (Waves, Ultrasound):ti,ab,kw OR (Pulsed Ultrasound):ti,ab,kw OR (Pulsed Ultrasounds):ti,ab,kw OR (Ultrasound, Pulsed):ti,ab,kw OR (Ultrasounds, Pulsed):ti,ab,kw OR (Low Intensity Pulsed Ultrasound):ti,ab,kw OR (LIPUS):ti,ab,kw OR (Low-Intensity Pulsed Ultrasound (LIPUS)):ti,ab,kw OR (Low Intensity Pulsed Ultrasound (LIPUS)):ti,ab,kw OR (Ultrasonic Diagnoses):ti,ab,kw OR (Low-Intensity Pulsed Ultrasounds (LIPUS)):ti,ab,kw OR (Pulsed Ultrasound, Low-Intensity (LIPUS)):ti,ab,kw OR (Pulsed Ultrasounds, Low-Intensity (LIPUS)):ti,ab,kw OR (Ultrasound, Low-Intensity Pulsed (LIPUS)):ti,ab,kw OR (Ultrasounds, Low-Intensity Pulsed (LIPUS)):ti,ab,kw OR (Low Intensity Pulsed Ultrasound Radiation):ti,ab,kw

#19 MeSH descriptor: [Ultrasonography, Doppler] explode all trees

#20 (Ultrasonography, Doppler):ti,ab,kw OR (Doppler Ultrasound):ti,ab,kw OR (Doppler Ultrasounds):ti,ab,kw OR (Ultrasound, Doppler):ti,ab,kw OR (Ultrasounds, Doppler):ti,ab,kw OR (Doppler Ultrasonography):ti,ab,kw OR (Doppler Ultrasound Imaging):ti,ab,kw OR (Doppler Ultrasound Imagings):ti,ab,kw OR (Imaging, Doppler Ultrasound):ti,ab,kw OR (Imagings, Doppler Ultrasound):ti,ab,kw OR (Ultrasound Imaging, Doppler):ti,ab,kw OR (Ultrasound Imagings, Doppler):ti,ab,kw

#21 #1 OR #2

#22 #3 OR #4 OR #5 OR #6 OR #7 OR #8 OR #9 OR #10 OR #11 OR #12

#23 #13 OR #14 OR #15 OR #16 OR #17 OR #18 OR #19 OR #20

#24 #21 AND #22 AND #23

EMBASE

#1 'radial artery'/exp

#2 'radial artery'/exp OR 'artery, radial':ab,ti OR 'arteria radialis':ab,ti OR 'radial arteria':ab,ti OR 'radialis artery':ab,ti

#3 'catheterization'/exp

#4 'catheterization':ab,ti OR 'catherization':ab,ti OR 'catheter detachment':ab,ti OR 'catheter technique':ab,ti OR 'catheterisation':ab,ti OR 'catheterisation, peripheral':ab,ti OR 'catheterization, peripheral':ab,ti OR 'microcatheterisation':ab,ti OR 'microcatheterization':ab,ti OR 'peripheral catheterisation':ab,ti OR 'peripheral catheterization':ab,ti

#5 'artery catheterization'/exp

#6 'artery catheterization':ab,ti OR 'arterial catheterisation':ab,ti OR 'arterial catheterization':ab,ti OR 'artery cannulation':ab,ti OR 'artery catheterisation':ab,ti OR 'catheterisation, artery':ab,ti OR 'catheterization, artery':ab,ti OR 'percutaneous arterial catheterisation':ab,ti OR 'percutaneous arterial catheterization':ab,ti

#7 'blood vessel catheterization'/exp

#8 'blood vessel catheterization':ab,ti OR 'blood vessel catheterisation':ab,ti OR 'vascular catheterisation':ab,ti OR 'vascular catheterization':ab,ti

#9 'vascular access device'/exp

#10 'vascular access device':ab,ti OR 'dialock':ab,ti OR 'vascular access devices':ab,ti OR 'vascular catheter introduction set':ab,ti OR 'vectra (vascular access device)':ab,ti

#11 'catheter'/exp

#12 'catheter':ab,ti OR 'aerostat':ab,ti OR 'catheter device':ab,ti OR 'catheter, device (physical object)':ab,ti OR 'catheters':ab,ti OR 'duplocath':ab,ti OR 'duplocath mis application catheter':ab,ti OR 'kumpe (catheter)':ab,ti

#13 'puncture'/exp

#14 'puncture':ab,ti OR 'punctures':ab,ti OR 'spinal tap':ab,ti

#15 'artery puncture'/exp

#16 'artery puncture':ab,ti OR 'arterial puncture':ab,ti

#17 'ultrasound'/exp

#18 'ultrasound':ab,ti OR 'phonophoresis':ab,ti OR 'radiation, ultrasonic':ab,ti OR 'sonication':ab,ti OR 'sonification':ab,ti OR 'ultra sound':ab,ti OR 'ultrashell':ab,ti OR 'ultrasonic':ab,ti OR 'ultrasonic energy':ab,ti OR 'ultrasonic irradiation':ab,ti OR 'ultrasonic measurement':ab,ti OR 'ultrasonic sound':ab,ti OR 'ultrasonic wave':ab,ti OR 'ultrasonic waves':ab,ti OR 'ultrasonics':ab,ti OR 'ultrasound radiation':ab,ti

#19 'echography'/exp

#20 'echography':ab,ti OR 'diagnostic ultrasonic examination':ab,ti OR 'diagnostic ultrasonic imaging':ab,ti OR 'diagnostic ultrasonic method':ab,ti OR 'diagnostic ultrasound':ab,ti OR 'doptone; duplex echography':ab,ti OR 'echogram':ab,ti OR 'echographic evaluation':ab,ti OR 'echoscopy':ab,ti OR 'echosound':ab,ti OR 'high resolution echography':ab,ti OR 'scanning, ultrasonic':ab,ti OR 'sonogram; sonographic examination':ab,ti OR 'sonographic screening':ab,ti OR 'sonography':ab,ti OR 'ultrasonic detection':ab,ti OR 'ultrasonic diagnosis':ab,ti OR 'ultrasonic echo':ab,ti OR 'ultrasonic examination':ab,ti OR 'ultrasonic scanning':ab,ti OR 'ultrasonic scintillation':ab,ti OR 'ultrasonogram':ab,ti OR 'ultrasonographic examination':ab,ti OR 'ultrasonographic screening':ab,ti OR 'ultrasonography':ab,ti OR 'ultrasound diagnosis':ab,ti OR 'ultrasound scanning':ab,ti

#21 'doppler ultrasonography'/exp

#22 'doppler ultrasonography':ab,ti OR 'doppler echography':ab,ti OR 'echography, doppler':ab,ti OR 'ultrasonography, doppler':ab,ti

#23 'doppler ultrasonography'/exp

#24 'ultrasound therapy':ab,ti OR 'therapeutic ultrasonic irradiation':ab,ti OR 'therapeutic ultrasonic radiation':ab,ti OR 'therapeutic ultrasonic stimulation':ab,ti OR 'therapeutic ultrasonic treatment':ab,ti OR 'therapeutic ultrasound':ab,ti OR 'therapeutic ultrasound radiation':ab,ti OR 'ultrasonic radiation therapy':ab,ti OR 'ultrasonic therapy':ab,ti OR 'ultrasound ablation':ab,ti

#25 'ultrasound therapy'/exp

#26 'pulsed doppler ultrasonography':ab,ti OR 'pulsed wave doppler ultrasonography':ab,ti OR 'ultrasonography, doppler, pulsed':ab,ti

#27 #1 OR #2

#28#3 OR #4 OR #5 OR #6 OR #8 OR #9 OR #10 OR #11 OR #12 OR #13 OR #14 OR #15 OR #16

#29 #17 OR #18 OR #19 OR #20 OR #21 OR #22 OR #23 OR #24 OR #25 OR #26

#30 #27 AND #28 AND #29

#31 'crossover procedure':de OR 'double-blind procedure':de OR 'randomized controlled trial':de OR 'single-blind procedure':de OR random*:de,ab,ti OR factorial*:de,ab,ti OR crossover*:de,ab,ti OR ((cross NEXT/1 over*):de,ab,ti) OR placebo*:de,ab,ti OR ((doubl* NEAR/1 blind*):de,ab,ti) OR ((singl* NEAR/1 blind*):de,ab,ti) OR assign*:de,ab,ti OR allocat*:de,ab,ti OR volunteer*:de,ab,ti

#32 #30 AND #31

Web Of Science

#1 TS=(radial artery OR artery, radial OR arteria radialis OR radial arteria OR radialis artery)

#2 TS=(catheterization OR catherization OR catheter detachment OR catheter technique OR catheter technique OR catheterisation OR catheterisation, peripheral OR catheterization, peripheral OR microcatheterisation OR microcatheterization OR peripheral catheterisation OR peripheral catheterization)

#3 TS=(artery catheterization OR Arterial catheterisation OR arterial catheterization OR artery cannulation OR artery catheterisation OR catheterisation, artery OR catheterization, artery OR percutaneous arterial catheterisation OR percutaneous arterial catheterization)

#4 TS=(blood vessel catheterization OR blood vessel catheterisation OR vascular catheterisation OR vascular catheterization)

#5 TS=(vascular access device OR Dialock OR vascular access devices OR vascular catheter introduction set OR Vectra (vascular access device) )

#6 TS=(Catheter OR AeroStat OR catheter device OR catheter, device (physical object) OR catheters OR Duplocath OR Duplocath MIS application catheter OR Kumpe (catheter) )

#7 TS=(puncture OR punctures OR spinal tap)

#8 TS=(artery puncture OR arterial puncture)

#9 TS=(Ultrasound OR phonophoresis OR radiation, ultrasonic OR sonication OR sonification ultra sound ultrashell ultrasonic OR ultrasonic energy OR ultrasonic irradiation OR ultrasonic measurement OR ultrasonic sound OR ultrasonic wave OR ultrasonic waves OR ultrasonics OR ultrasound radiation)

#10 TS=(echography OR diagnostic ultrasonic examination OR diagnostic ultrasonic imaging OR diagnostic ultrasonic method OR diagnostic ultrasound OR doptone; duplex echography OR echogram OR echographic evaluation OR echoscopy OR echosound OR high resolution echography OR scanning, ultrasonic OR sonogram; sonographic examination OR sonographic screening OR sonography OR ultrasonic detection OR ultrasonic diagnosis OR ultrasonic echo OR ultrasonic examination OR ultrasonic scanning OR ultrasonic scintillation OR ultrasonogram OR ultrasonographic examination OR ultrasonographic screening OR ultrasonography OR ultrasound diagnosis OR ultrasound scanning)

#11 TS=(Doppler ultrasonography OR Doppler echography OR echography, Doppler OR ultrasonography, Doppler)

#12 TS=(ultrasound therapy OR therapeutic ultrasonic irradiation OR therapeutic ultrasonic radiation OR therapeutic ultrasonic stimulation OR therapeutic ultrasonic treatment OR therapeutic ultrasound OR therapeutic ultrasound radiation OR ultrasonic radiation therapy OR ultrasonic therapy OR ultrasound ablation)

#13 TS=(pulsed Doppler ultrasonography OR pulsed wave Doppler ultrasonography OR ultrasonography, Doppler, pulsed)

#14 #8 OR #7 OR #6 OR #5 OR #4 OR #3 OR #2

#15 #13 OR #12 OR #11 OR #10 OR #9

#16 #15 AND #14 AND #1

#17 TS= clinical trial* OR TS=research design OR TS=comparative stud* OR TS=evaluation stud* OR TS=controlled trial* OR TS=follow-up stud* OR TS=prospective stud* OR TS=random* OR TS=placebo* OR TS=(single blind*) OR TS=(double blind*)

#18 #17 AND #16

**Supplement 2.** Risk of bias assessment for included studies

**
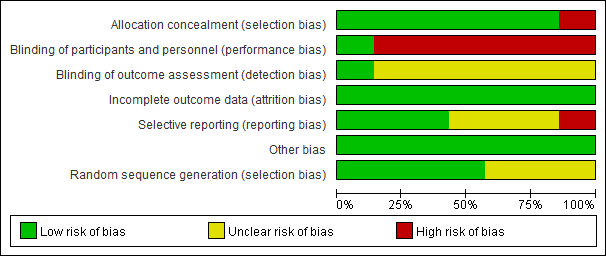
**

**
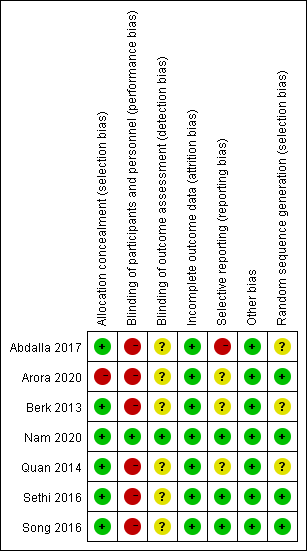
**

**Supplement 3.** The GRADE evidence profile for ultrasound-guided radial arterial cannulation.

|  | Risk of bias | Inconsistency | Indirectness | Imprecision | Publication bias | Rating up situations^a^ | Relative (95% CI) | Quality |
| --- | --- | --- | --- | --- | --- | --- | --- | --- |
| The rate of cannula insertion success on the first attempt | No | Serious^b^ | No | No | Undected | None | RR 1.03 (0.83 to 1.28) | MODERATE |
| Total time to successful cannulation | No | Serious^b^ | No | No | Undected | None | MD 3.9 lower (18.3 lower to 10.49 higher) | MODERATE |
| Final success rate | No | No | No | No | Strongly suspected^c^ | None | RR 0.99 (0.96 to 1.02) | MODERATE |
| Number of cannulation attempts | No | Serious^b^ | No | No | Undected | None | MD 0.33 lower (0.52 to 0.14 lower) | MODERATE |
| Ultrasonic location time | No | Serious^b^ | No | No | Undected | None | MD 13.67 lower (18.7 to 8.63 lower) | MODERATE |
| Posterior wall damage | No | Serious^b^ | No | Serious^d^ | Undected | None | RR 2.04 (0.64 to 6.54) | LOW |

*a.* *Rating up situations included large effect, dose response gradient, and direction of plausible confounding.*

*b.High I^2^ and non-overlapping confidence intervals suggest important inconsistency which lowers our certainty in effect.*

*c.Because of result from Egger's test (P=0.037), we downgrade the quality of evidence by one level.*

*d.Point estimate and 95% confidence intervals don’t exclude important benefit and important harm.*

**Supplement 4.** Sensitivity analysis

|  | The rate of cannula insertion success on the first attempt | | Total time to successful cannulation | | Final success rate | | Number of cannulation attempts | | Ultrasonic Location time | | Posterior wall damage | |
| --- | --- | --- | --- | --- | --- | --- | --- | --- | --- | --- | --- | --- |
| Age(adult only) | Total | Adjusted | Total | Adjusted | Total | Adjusted | Total | Adjusted | Total | Adjusted | Total | Adjusted |
|  | 1.03 [0.83, 1.28] | 1.03 [0.81, 1.31] | -3.90 [-18.30, 10.49] | -1.88 [-16.95, 13.20] | 0.99 [0.96, 1.02] | 0.99 [0.96, 1.03] | -0.01 [-0.30, 0.27] | NA | -4.80 [-5.35, -4.25] | NA | 2.04 [0.64, 6.54] | 1.14 [0.18, 7.23] |
| Effect measures | RR | OR | MD | SMD | RR | OR | MD | SMD | MD | SMD | RR | OR |
|  | 1.03 [0.83, 1.28] | 1.23 [0.53, 2.86] | -1.88 [-16.95, 13.20] | -0.24 [-0.95, 0.47] | 0.99 [0.96, 1.02] | 0.76 [0.39, 1.45] | -0.01 [-0.30, 0.27] | -0.00 [-0.49, 0.49] | -13.67 [-18.70, -8.63] | -1.90 [-2.83, -0.98] | 2.04 [0.64, 6.54] | 5.68 [0.37, 87.07] |
| Analysis methods | Random | Fixed | Random | Fixed | Random | Fixed | Random | Fixed | Random | Fixed | Random | Fixed |
|  | 1.03 [0.83, 1.28] | 1.05 [0.97, 1.15] | -1.88 [-16.95, 13.20] | -2.46 [-4.43, -0.50] | 1.00 [0.98, 1.02] | 0.99 [0.96, 1.02] | -0.01 [-0.30, 0.27] | -0.02 [-0.11, 0.07] | -13.67 [-18.70, -8.63] | -5.89 [-6.38, -5.40] | 2.04 [0.64, 6.54] | 2.82 [1.97, 4.04] |

**Supplement** 5**.** Begg’s and Egger’s tests for the primary outcome

Begg's Test

adj. Kendall's Score (P-Q) = 1

Std. Dev. of Score = 6.66

Number of Studies = 7

z = 0.15

Pr > |z| = 0.881

z = 0.00 (continuity corrected)

Pr > |z| = 1.000 (continuity corrected)

Egger's test

------------------------------------------------------------------------------

Std_Eff | Coef. Std. Err. t P>|t| [95% Conf. Interval]

-------------+----------------------------------------------------------------

slope | .1949218 .2796886 0.70 0.517 -.5240407 .9138842

bias | -1.231875 2.56399 -0.48 0.651 -7.822822 5.359072

------------------------------------------------------------------------------

**Supplement 6.** Summary of the meta-analyses

|  | Type of outcomes | Number of patients(male/female) | Incidence rate | Variable type | Effect measures | Statistic Effect/measure Effect |
| --- | --- | --- | --- | --- | --- | --- |
| the rate of cannula insertion success on the first attempt | Primary outcome | 826（414/412） | 72.46%/68.69% | Dichotomous | Random effect | RR |
| Total time to successful cannulation | Secondary outcome | 826（414/412） | / | Continuous | Random effect | MD/WMD |
| Final success rate | Secondary outcome | 634（318/316） | 93.72%/94.90% | Dichotomous | Fixed effect | RR |
| Number of cannulation attempts | Secondary outcome | 575（289/286） | / | Continuous | Fixed effect | MD/WMD |
| Ultrasonic location time | Secondary outcome | 550（276/274） | / | Continuous | Random effect | MD/WMD |
| Posterior wall damage | Secondary outcome | 345（174/171） | 47.13%/16.96% | Dichotomous | Random effect | RR |
| Thrombosis | Secondary outcome | 136（70/66） | 0%/1.52% | Dichotomous | Random effect | OR |
| Edema | Secondary outcome | 108（54/54） | 12.96%/0 | Dichotomous | Random effect | OR |
| Hematoma | Secondary outcome | 725（364/361） | 16.48%/9.70% | Dichotomous | Random effect | RR |
| Vasospasm | Secondary outcome | 394（199/195） | 4.02%/5.64% | Dichotomous | Random effect | OR |
| Depth from skin/ Skin-to-artery distance | Secondary outcome | 742（372/370） | / | Continuous | Random effect | MD/WMD |
| Diameter of radial artery/ Antero-posterior arterial diameter | Secondary outcome | 742（372/372） | / | Continuous | Fixed effect | MD/WMD |

Summary of the meta-analyses(continued)

I² describes the heterogeneity

|  | Effect quantity | P | 95% CI | I² | Begg's Test | Egger's test |
| --- | --- | --- | --- | --- | --- | --- |
| the rate of cannula insertion success on the first attempt | 1.03 | 0.79 | [0.83, 1.28] | 83.00% | 1 | 0.651 |
| Total time to successful cannulation | -3.9 | 0.6 | [-18.30, 10.49] | 97.00% | 1 | 0.784 |
| Final success rate | 0.99 | 0.42 | [0.96, 1.02] | 0.00% | 0.221 | 0.037 |
| Number of cannulation attempts | -0.01 | 0.93 | [-0.30, 0.27] | 89.00% | 1 | 0.901 |
| Ultrasonic location time | -13.67 | 0 | [-18.70, -8.63] | 97.00% | 1 | 0.141 |
| Posterior wall damage | 2.04 | 0.23 | [0.64, 6.54] | 87.00% | 1 | 0.299 |
| Thrombosis | 0.31 | 0.48 | [0.01, 7.74] | / | / | / |
| Edema | 17.21 | 0.05 | [0.96, 309.37] | / | / | / |
| Hematoma | 2.27 | 0.21 | [0.63, 8.09] | 78.00% | 0.452 | 0.189 |
| Vasospasm | 1.52 | 0.75 | [0.11, 20.24] | 71.00% | 1 | 0.056 |
| Depth from skin/ Skin-to-artery distance | -0.04 | p = 0.77 | [-0.30, 0.22] | 67.00% | 1 | 0.716 |
| Diameter of radial artery/ Antero-posterior arterial diameter | -0.01 | 0.88 | [-0.10, 0.09] | 17.00% | 0.221 | 0.136 |

**Supplement 7.** The sensitivity analysis of primary outcome


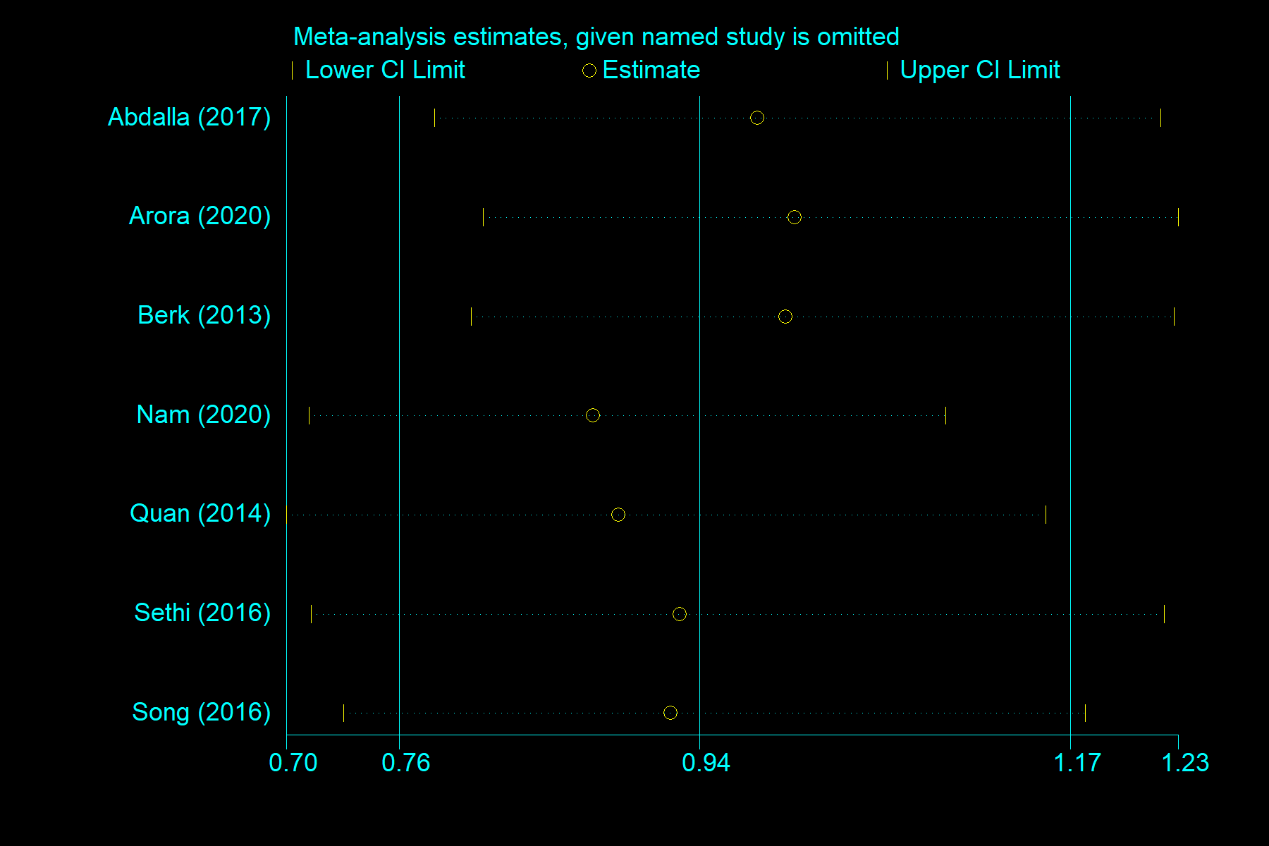


**Supplement 8.** Subgroup analysis based on operation experience –SA-OOP vs LA-IP

1. the rate of cannulation on the first attempt


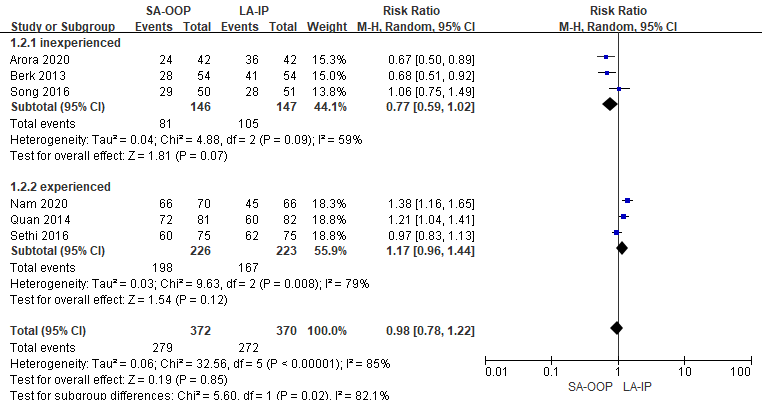


1. final success rate


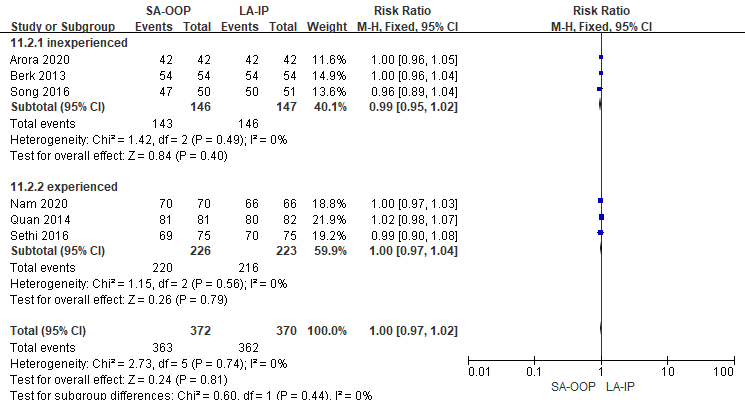


1. total time to successful cannulation


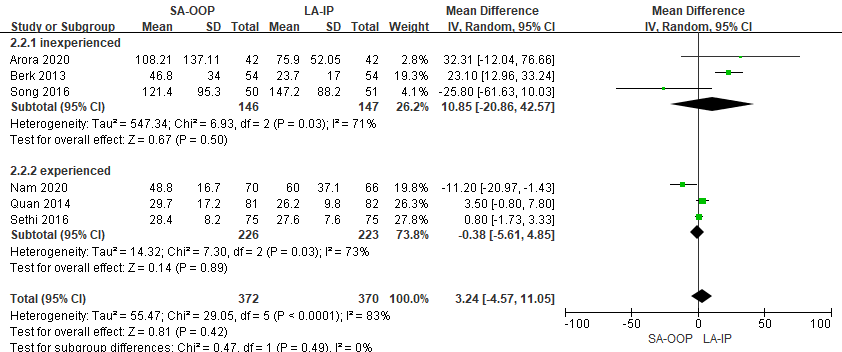


D. number of cannulation attempts


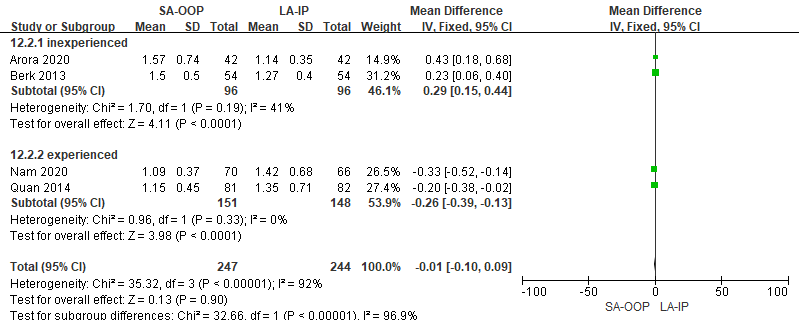


E. ultrasonic location time


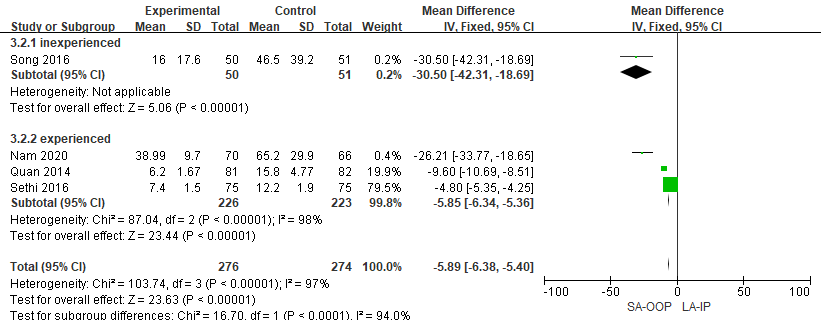


F. posterior wall damage


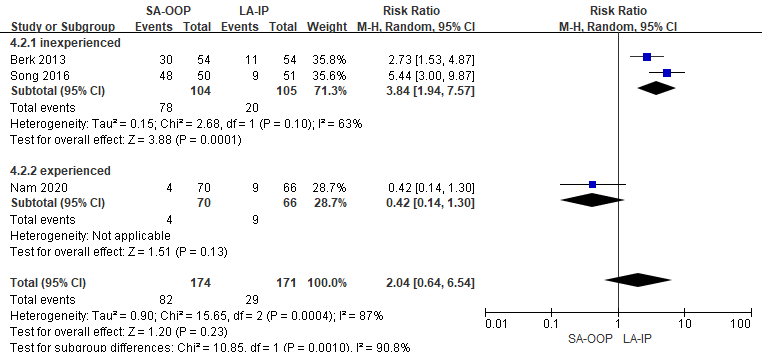


G. hematoma


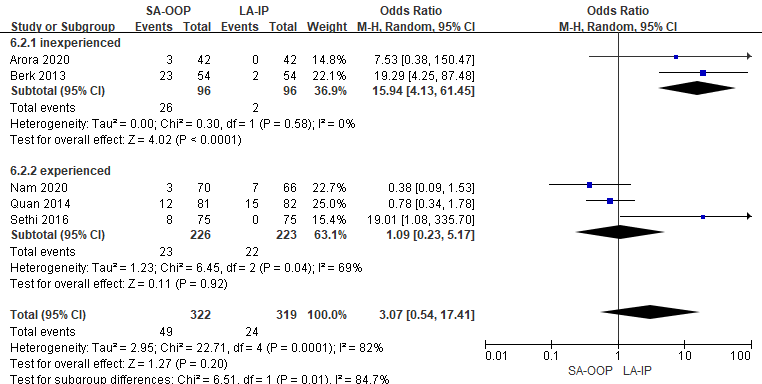


H. vasospasm


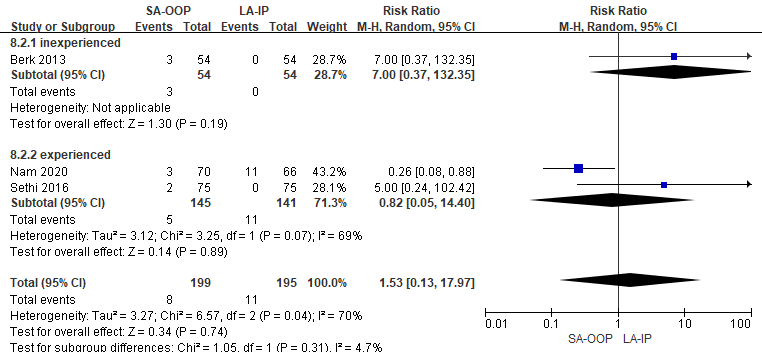


**Supplement 9.** Forrest and funnel plots for secondary outcomes

A. total time to successful cannulation.


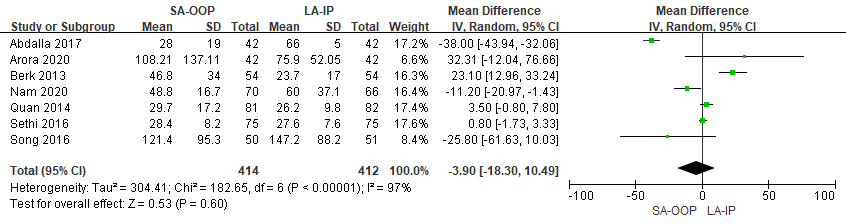


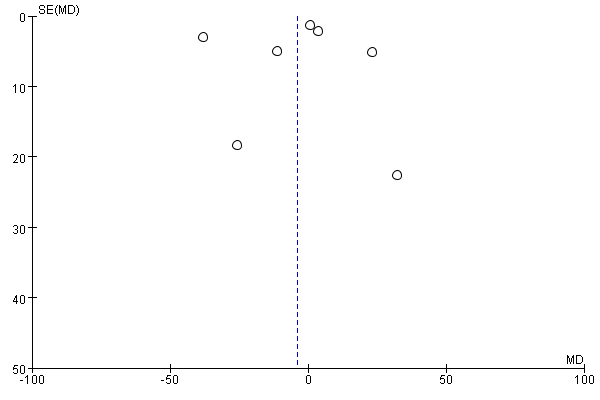


B. final success rate.


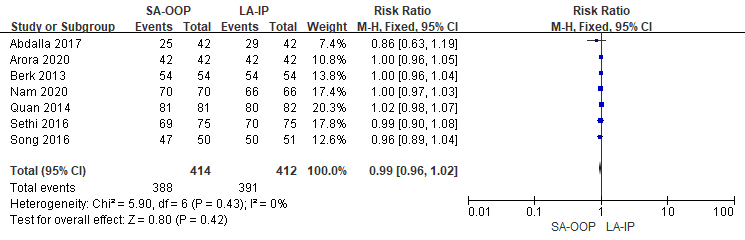


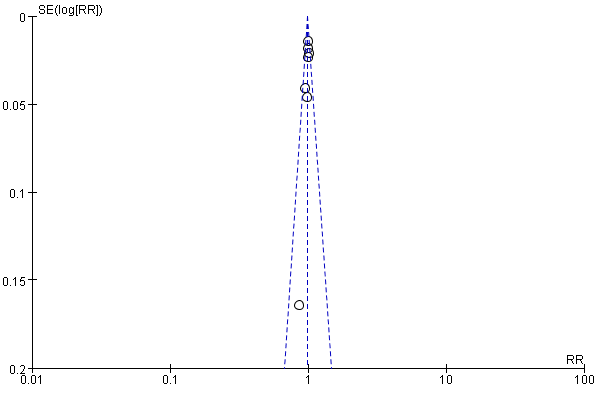


C. Number of cannulation attempts


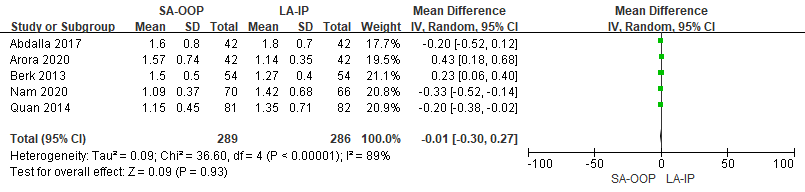


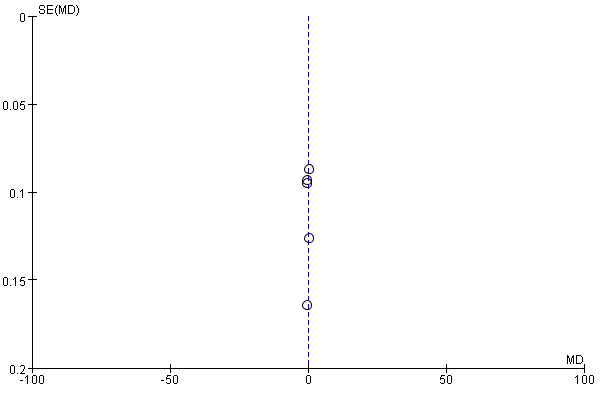


D. ultrasonic location time


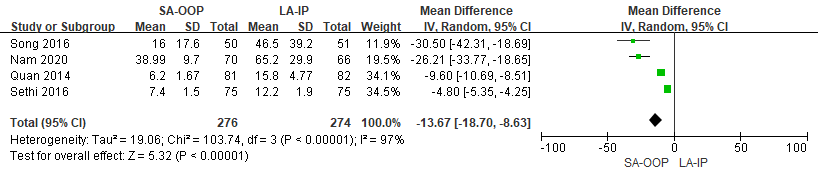


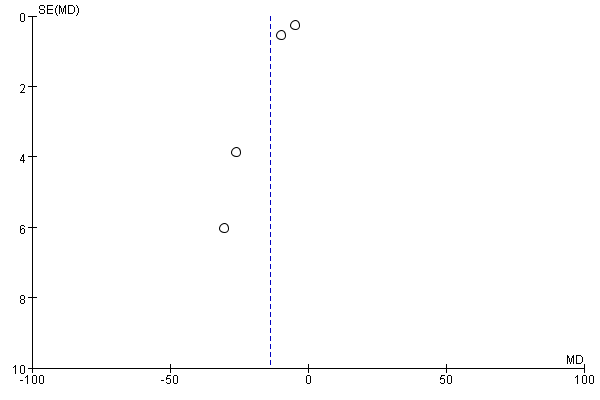


E. posterior wall damage.


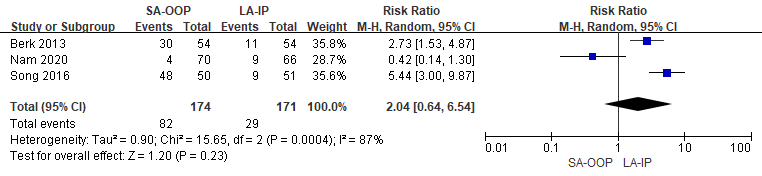


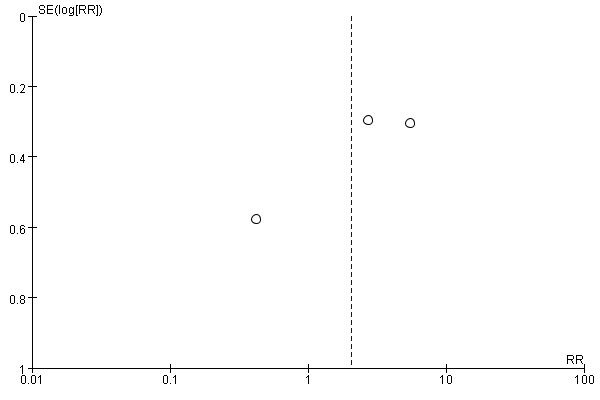


F.thrombosis


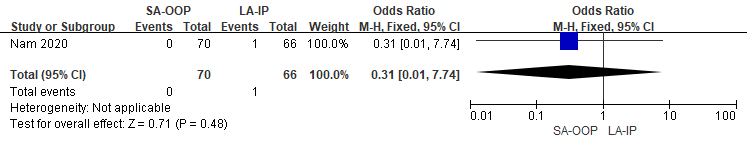


G. hematoma


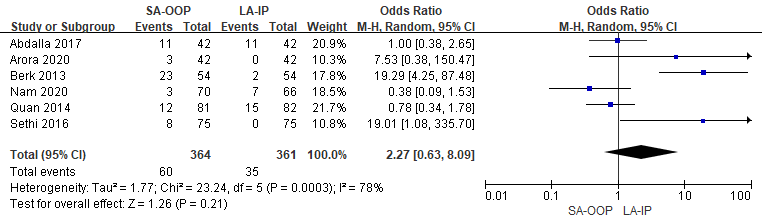


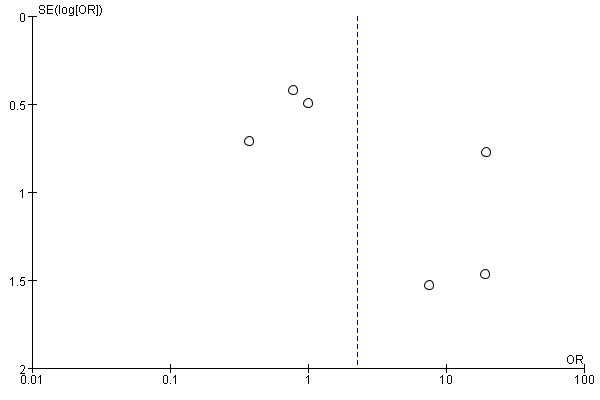


H.edema


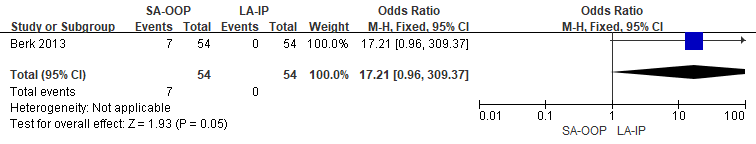


I.vasospasm


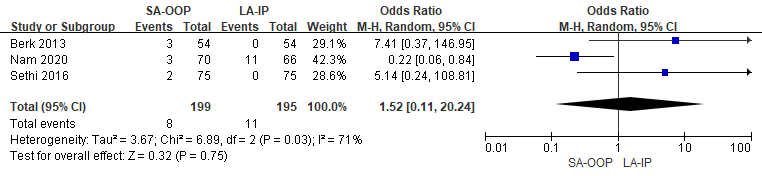


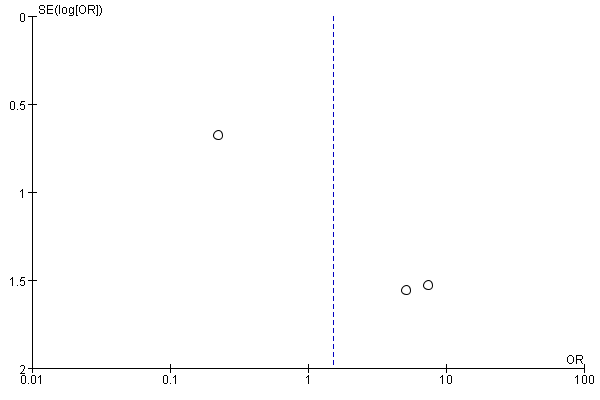


J.depth from skin


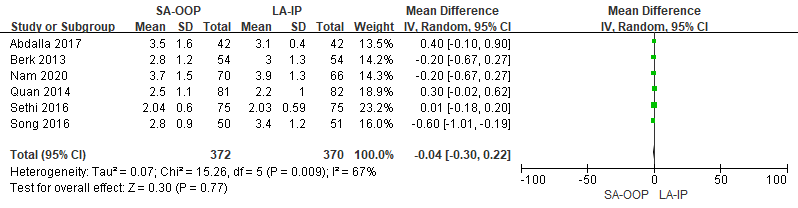


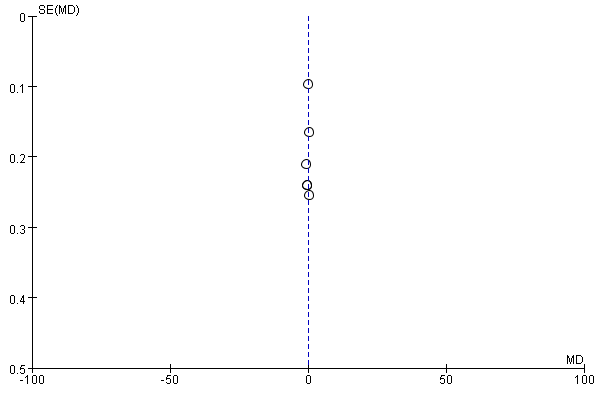


K.diameter of radial artery


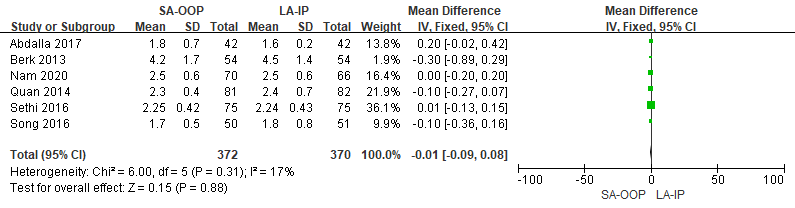


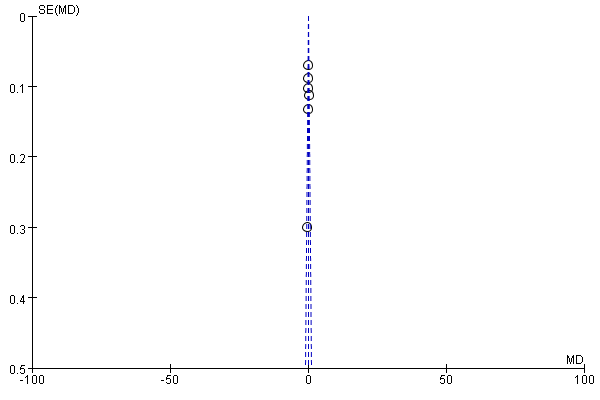


**Supplement 10.** Forrest and funnel plots for primary outcomes

The rate of cannulation on the first attempt

– short-axis out of plant group vs long-axis in plant group.


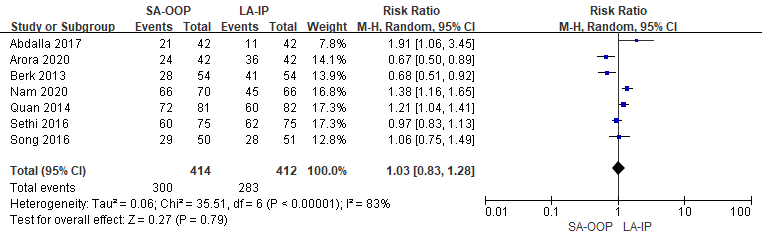


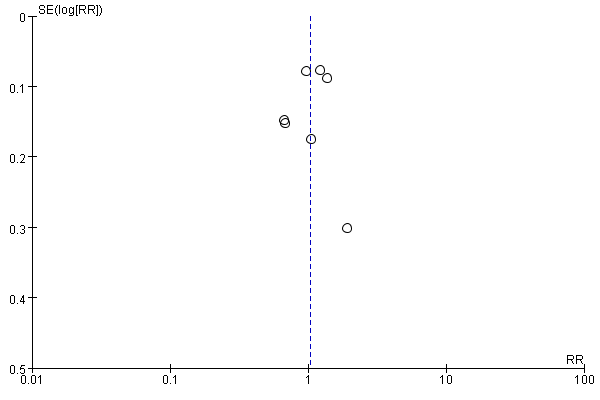

Supplement: Supplementary file 1 [file Data_Sheet_1.docx]
